# Effect of the Dodecanoate Anion on Thermal Stability and Decomposition Mechanism of Mono- and Dicationic Ionic Liquids

**DOI:** 10.1021/acsomega.4c10596

**Published:** 2025-02-26

**Authors:** Jean C. B. Vieira, Marcos A. Villetti, Caroline R. Bender, Clarissa P. Frizzo

**Affiliations:** † NUQUIMHE, Department of Chemistry, Federal University of Santa Maria, Santa Maria 97105-900, Brazil; ‡ LEPOL, Department of Physics, Federal University of Santa Maria, Santa Maria 97105-900, Brazil

## Abstract

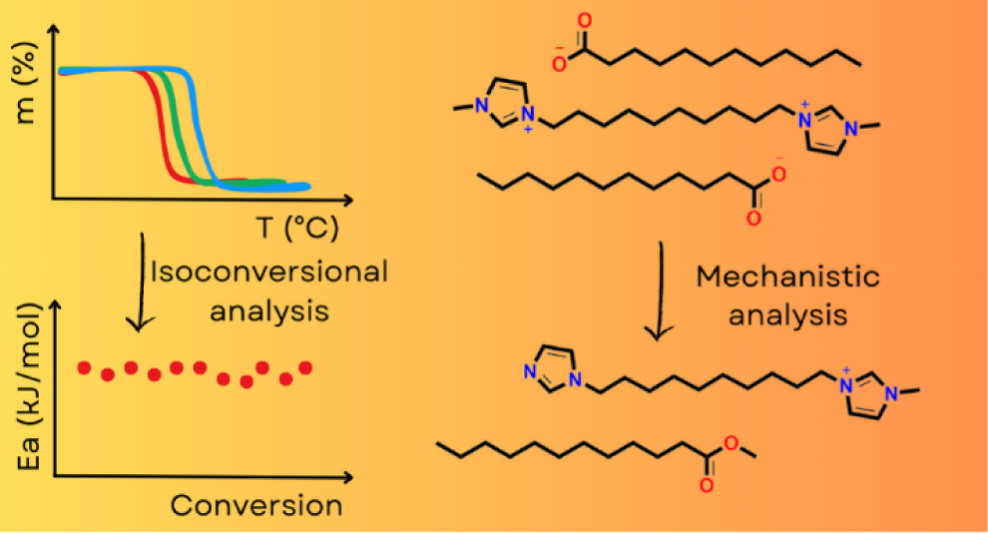

This work presents the synthesis of mono- and dicationic ionic liquids (ILs) that combine the cations 1-butyl-3-methylimidazolium ([C_4_MIM]^+^), 1-decyl-3-methylimidazolium ([C_10_MIM]^+^), 1,4-bis(3-methylimidazolium-1-yl)butane ([C_4_(MIM)_2_]^2+^), and 1,10-bis(3-methylimidazolium-1-yl)decane ([C_10_(MIM)_2_]^2+^) with the anion dodecanoate ([C_11_COO]^−^), along with a study into their thermal stability and mechanism for thermal decomposition. Thermal stability was investigated using the Kissinger–Akahira–Sunose (KAS) isoconversional method to determine the isoconversional activation energies (E*_α_*) and compensation effect to calculate the pre-exponential factor (ln *A*_*α*_). The results showed that there was no significant difference in the thermal stabilities between the ILs, with all compounds being thermally stable up to 450 K. The thermal decomposition mechanism was analyzed using nuclear magnetic resonance (NMR), electrospray ionization mass spectrometry (ESI-MS), and thermogravimetric analysis coupled with Fourier-transform infrared spectroscopy (TGA-FTIR). The main decomposition pathways were nucleophilic substitution at the lateral or spacer chain and the methyl group.

## Introduction

Ionic liquids (ILs) are organic salts composed of organic cations and either organic or inorganic anions that act as liquids at relatively low temperatures. These compounds are known to present a series of interesting properties, such as low vapor pressure, high thermal and electrical stabilities, nonflammability, and high electrical conductivity.^1,2^ They can be used as catalysts,^3^ solvents for chemical reactions,^4,5^ and for separation processes^6,7^ and are one of the few classes of solvents that can dissolve biopolymers, such as cellulose.^8−10^ The great variety of applications for ILs comes from the nearly unlimited number of possible combinations of different cations and anions. For example, the presence of long alkyl chains in the IL structure imparts surfactant properties.^11,12^ ILs with an additional positive charge in the cation are known as dicationic ionic liquids (DILs) and exhibit increased thermal stability and decreased toxicity than their monocationic analogs, which is advantageous considering applications in high-temperature processes, in addition to a lower environmental impact.^13−18^ Moreover, DILs have been used as stationary phases in chromatography^19−22^ and as lubricants^23,24^ and surfactants.^25−27^

The thermal stability of DILs increases compared to their monocationic analogs because the introduction of a second cationic moiety leads to an increase in hydrogen bonding and electrostatic interactions between cation and anion, thus providing additional modes of thermal energy storage.^28,29^ In addition, the second cationic head leads to an increase in molecular symmetry and consequent higher lattice energy, making the decomposition process more energy-intensive.^30^ The lower toxicity of DILs can be attributed to a decrease in lipophilicity compared to their monocationic analogs, since one of the main mechanisms of cellular incompatibility of ILs is the interaction with the cellular membrane leading to rupture.^18^

The properties of ILs are also strongly influenced by the structure of the anion. Carboxylate-containing ILs have attracted increasing interest because they can be generated from environmentally friendly carboxylic acids or amino acids.^14,31−34^ Recently, carboxylate ILs with anions derived from long alkyl chain carboxylic acids have been reported, with primary focus on their use as lubricants.^35−38^ These ILs form tribofilms on the surfaces of lubricated parts, which reduce the amount of friction and wear in the system.^38^ When considering the application of an IL as a lubricant, it is important to study its thermal stability because the movement of the lubricated parts of any system can generate heat and, therefore, lead to thermal decomposition.^37^

Thermal stability is commonly evaluated using thermogravimetric analysis (TGA),^39^ enabling experimental determination of short-term thermal stability, obtained from single and rapid experiments and mainly expressed in terms of onset temperatures, and long-term thermal stability, which requires more complex analysis such as isothermal mass loss experiments or multiple heating rate experiments. Short-term thermal stability, however, is generally overestimated by TGA; therefore, a more appropriate approach is to perform a long-term thermal stability study entailing a kinetic analysis of thermal decomposition.^40,41^ The kinetic analysis of thermal decomposition is mainly aimed at providing the Arrhenius parameters, i.e., activation energy (*E*_a_) and pre-exponential factor (*A*) for the decomposition process under investigation, allowing a better understanding of the whole process, including information about the mechanisms of thermal decomposition.^42^

There are two possible approaches when performing a kinetic analysis using TGA, the first one using multiple isothermal experiments at different temperatures while the second approach uses multiple heating rates in dynamic experiments.^42^ The multiple heating rates methodology is called isoconversional and is considered to be the preferable approach to the kinetic analysis using TGA data.^40,41^ Recently, our research group has studied the kinetics of the thermal decomposition of several mono- and dicationic carboxylate ILs using a isoconversional methodology and found they exhibit lower thermal stability than their halogenated analogs,^43,44^ limiting the temperature range in which these carboxylate ILs may be applied. The ILs investigated were composed of butanoate ([C_4_H_7_O_2_]^−^) and heptanoate ([C_7_H_13_O_2_]^−^) anions, which could be considered to be small- and medium-sized, respectively. These findings raise the question of whether the use of even bulkier anions, such as dodecanoate ([C_12_H_23_O_2_]^−^), may be a potentially viable strategy to achieve improved thermal stability compared to the previously studied ILs, thus extending the operating temperature of carboxylate imidazolium-based ILs.

In addition to establishing a thermal stability order, studying the thermal decomposition mechanism of ILs is important to understand how the cation and anion structures influence which decomposition products are formed. This knowledge allows the design of new ILs with improved thermal stability targeting applications in high-temperature processes.^45−47^ The decomposition mechanisms of mono- and dicationic imidazolium-based ILs have been studied before. The main reaction pathways include nucleophilic substitution at the positions adjacent to the imidazolium ring and the deprotonation of the acidic hydrogen in position 2 (between the nitrogen atoms).^47,48^ These mechanisms, however, have been mainly studied considering inorganic anions or relatively small carboxylates^43,44,49^ so the influence of bulkier carboxylates such as dodecanoate ([C_12_H_23_O_2_]^−^) remains to be fully investigated.

**Figure 1:**
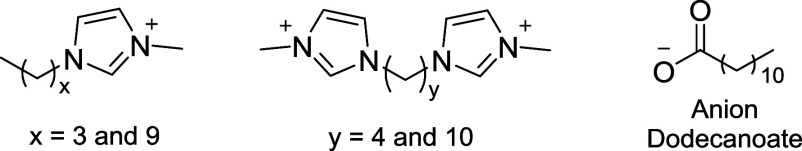
Chemical structures of the cations and anion of the ILs reported in this work.

Considering the growing interest in ILs with long alkyl chain carboxylates, their potential applications and the need to evaluate their thermal stability, the objectives of the present study were: (1) to synthesize mono- and dicationic ILs by combining the dodecanoate anion, [C_11_COO]^−^, and the cations 1-butyl-3-methylimidazolium ([C_4_MIM]^+^), 1-decyl-3-methylimidazolium ([C_10_MIM]^+^), 1,4-bis(3-methylimidazolium-1-yl)butane ([C_4_(MIM)_2_]^2+^), and 1,10-bis(3-methylimidazolium-1-yl)decane ([C_10_(MIM)_2_]^2+^); (2) determine their thermal stability using an isoconversional approach; and (3) determine the mechanism for thermal decomposition. The chemical structures of the cations and anions used to form the ILs studied herein are shown in Figure 1.

## Experimental Details

### Materials

1-Methylimidazole, 1-bromobutane, 1-bromodecane, 1,4-dibromobutane, 1,10-dibromodecane, dodecanoic acid (Sigma–Aldrich, USA), sodium hydroxide (Neon, Brazil), ethanol (Synth, Brazil), acetonitrile (Avantor, Inc., USA), and methanol (Merck, Germany) were used without further purification. The Amberlite IRN-78 OH (Sigma-Aldrich, USA) resin was used in the ion-exchange reactions.

### Synthesis

The procedures for synthesizing the mono- and dicationic dodecanoate ionic liquids were based on previous studies by our research group.^43,44,50^ First, precursor ILs with bromide anions were synthesized by reacting 1-methylimidazole with the respective alkyl halide with the appropriate stoichiometry. Subsequently, the bromide ILs were solubilized in ethanol (20 mL) and very slowly passed through a column containing an Amberlite IRN-78 OH ion-exchange resin (10 g). The amount of bromide ILs used was calculated based on the synthesis of 1 g of dodecanoate IL. An appropriate amount of dodecanoic acid was added to a round-bottomed flask containing the hydroxide IL ethanolic solution and stirred at room temperature for 48 h. The solution was then poured into a beaker, and the solvent was slowly evaporated using a hot plate. The dodecanoic ILs were purified by stirring overnight with ethyl acetate (50 mL), separated by decantation, and dried under reduced pressure.

#### 1-Butyl-3-methylimidazolium Dodecanoate ([C_4_MIM][C_11_COO]) and 1-Decyl-3-methylimidazolium Dodecanoate ([C_10_MIM][C_11_COO])

Hydrogen Nuclear Magnetic Resonance (^1^H NMR) spectra were consistent with those reported in the literature.^51,52^

#### 1,4-Bis(3-methylimidazolium-1-yl)butane Dodecanoate ([C_4_(MIM)_2_][C_11_COO]_2_)

C_36_H_66_N_4_O_4_^1^H NMR (CDCl_3_, 600 MHz, 298 K): δ_H_ (ppm) 10.57 (s, 2H, C*H*), 7.76 (s, 2H, C*H*), 7.25 (s, 2H, C*H*), 4.40 (t, 4H, C*H*_*2*_), 3.96 (s, 6H, C*H*_*3*_), 2.14 (t, 4H, C*H*_*2*_), 1.99 (quint, 4H, C*H*_*2*_), 1.57 (quint, 4H, C*H*_*2*_), 1.31–1.24 (m, 32H, C*H*_*2*_), 0.87 (t, 6H, C*H*_*3*_). ^13^C NMR (CDCl_3_, 150 MHz): δ_C_ (ppm) 180.38 (*C*O), 139.16 (*C*H), 122.84 (*C*H), 122.76 (*C*H), 48.51 (*C*H_2_), 39.14 (*C*H_2_), 36.18 (*C*H_3_), 31.93, 30.09, 29.78, 29.76, 29.70, 29.39, 27.14, 26.28, 22.70 (*C*H_2_), 14.13 (*C*H_3_). FTIR main bands (cm^–1^): 1556 (ν_as_ COO^–^), 1392 (ν_s_ COO^–^), 1169 (ν_as_ CH_3_–(N–C–N)^+^–CH_2_). Appearance: Colorless, pasty, solid. Yield: 97%.

#### 1,10-Bis(3-methylimidazolium-1-yl)decane Dodecanoate ([C_10_(MIM)_2_][C_11_COO]_2_)

C_42_H_78_N_4_O_4_^1^H NMR (CDCl_3_, 600 MHz, 298 K): δ_H_ (ppm) 11.34 (s, 2H, C*H*), 7.24 (s, 2H, C*H*), 7.23 (s, 2H, C*H*), 4.29 (t, 4H, C*H*_*2*_), 4.06 (s, 6H, C*H*_*3*_), 2.20 (t, 4H, C*H*_*2*_), 1.87 (quint, 4H, C*H*_*2*_), 1.63 (quint, 4H, C*H*_*2*_), 1.30–1.24 (m, 44H, C*H*_*2*_), 0.87 (t, 6H, C*H*_*3*_). ^13^C NMR (CDCl_3_, 150 MHz): δ_C_ (ppm) 180.72 (*C*O), 140.79 (*C*H), 122.65 (*C*H), 121.09 (*C*H), 49.79 (*C*H_2_), 39.35 (*C*H_2_), 36.27 (*C*H_3_), 31.93, 30.11, 29.75, 29.69, 29.38, 28.80, 28.59, 27.23, 25.94, 22.69 (*C*H_2_), 14.14 (*C*H_3_). FTIR main bands (cm^–1^): 1556 (ν_as_ COO^–^), 1392 (ν_s_ COO^–^), 1188 (ν_as_ CH_3_–(N–C–N)^+^–CH_2_). Appearance: White solid. Yield: 98%.

### Nuclear Magnetic Resonance (NMR) Analysis

All NMR experiments were performed using a Bruker Avance III NMR spectrometer operating at 600.13 MHz for ^1^H and 150.32 MHz for ^13^C. Approximately 15 mg of the ILs were solubilized in 500 μL of deuterated solvent in 5 mm NMR tubes, and the spectra were recorded at 298 K. The spectra were calibrated in relation to tetramethylsilane (TMS; δ_H_ = 0 ppm) when using CDCl_3_, and in relation to the residual solvent (δ_H_ = 2.50 ppm) when using DMSO-*d*_6_, according to the literature.^53^

### Fourier Transform Infrared Spectroscopy (FTIR) Analysis

Fourier transform infrared (FTIR) spectra were obtained using a Bruker Vertex 70 spectrometer equipped with an ATR sampling accessory. Sixty-four scans were acquired in the 4000 to 30 cm^–1^ region at a resolution of 4 cm^–1^. The data were exported and analyzed using OriginPro 9.5 (Northampton, MA, USA).

### Thermogravimetric Analysis (TGA)

The TGA experiments were performed using a TGA Q5000 instrument (TA Instruments Inc., USA). The equipment usage condition was accessed by measuring the mass loss of a sample of well-known thermal decomposition profile (CaC_2_O_4_.H_2_O 99.9%). The sample mass used for experiments involving multiple heating rates was in the range of 2–3 mg. For analyzing the decomposition mechanism, the sample mass used was in the range of 5–10 mg. All samples were subjected to a 30 min isotherm at 353 K prior to heating in order to eliminate volatiles. The heating rates used in the multiple heating rate experiments were 2, 5, 10, 15, and 20 K min^–1^ up to 673 K. For the decomposition mechanism analysis, experiments were performed with a heating rate of 10 K min^–1^ up to 493 K, then the residual material remaining in the crucible was cooled, solubilized in CDCl_3_ and analyzed by NMR. For the electrospray ionization mass spectrometry (ESI-MS) experiments, a small amount of the residue was dissolved in 1 mL of methanol for HPLC analysis. In all TGA experiments, a 25 mL min^–1^ N_2_ flux was applied to the samples. The obtained data was analyzed using OriginPro 9.5 (Northampton, MA, USA).

### Electrospray Ionization Mass Spectrometry (ESI-MS) Analysis

Mass spectra were obtained using a 6460 Triple Quadrupole Mass Spectrometer (Agilent Technologies, USA) operating in positive ion mode. Nitrogen was used as both the nebulization and collision gas. The gas temperature was 598 K, the flow of the drying gas was 0.5 mL min^–1^, and the nebulizer was set to 35 psi. The capillary voltage was 4500 V, and the fragmentor voltage was 0 eV.

### TGA-FTIR

Thermogravimetric analysis coupled with Fourier-transform infrared spectroscopy was performed using an SDT Q600 analyzer (TA Instruments Inc., USA) coupled to a Nicolet 6700 spectrophotometer (Thermo Fischer Scientific, USA). The samples were heated at a rate of 10 K min^–1^ from room temperature to 673 K, and the N_2_ flow rate was set at 100 mL min^–1^.

### Kinetic Study

An investigation into the kinetic parameters associated with thermal decomposition, including the extent of decomposition, is crucial for improving the reliability of thermal stability profiles for ILs. In addition, the analysis performed herein provided insight into the mechanism for thermal decomposition. These findings are attainable when deploying isoconversional kinetic analysis based on experiments with multiple heating rates.^42^ In this study, we used the Kissinger–Akahira–Sunose (KAS) methodology, as recommended by the ICTAC Kinetics Committee.^40,41^

First, the TGA curves were converted into temperature (T) versus extent of conversion (α) curves using eq 1, where m*_i_*, m*_T_*, and m*_f_* are the masses at the beginning, at temperature T (in Kelvin), and at the end of the experiment, respectively. Then, at defined α values from 0.1 to 0.9 in 0.05 increments, the temperature values were applied using the KAS method (eq 2),^54^ where q is the heating rate and R is the gas constant. From the slope of the ln(q/T^2^) versus 1/T curve, it is possible to calculate a value for isoconversional activation energy (E*_α_*) for each value of α.
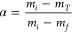
1
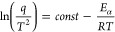
2

The kinetic profile of a given thermally stimulated process can be described in terms of conversion by three parameters – E*_α_*, the pre-exponential factor (*A*_*α*_) and *f(α)*.^42^ The pre-exponential factor, *A*_*α*_, can be calculated using the compensation effect if the thermal decomposition is a single-step process. This approach considers a set of E*_αj_* and *ln A*_*αj*_ values calculated using a single-step kinetic equation and a series of known kinetic models. These values have no physical meaning; however, they have a linear relationship, as expressed in eq 3. Knowing the values of a and b allows for *ln A*_*α*_ to be calculated by substituting E*_α_* into eq 3 in order for meaningful *ln A*_*α*_ values to be calculated using the isoconversional method. A more detailed explanation of the compensation effect can be found in literature.^42,55^
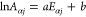
3

After calculating both the E*_α_* and *ln A*_*α*_ values it is possible to determine the profile of the kinetic model (*f(α)*) curve, which gives insight into the mechanism for thermal decomposition. These three kinetic parameters are correlated, as expressed in eq 4, which upon isolation of *f(α)*, can be converted into eq 5, which enables a value of *f(α)* to be calculated for each value of α considered in this study.^42,56^
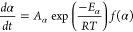
4
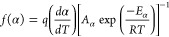
5

The errors in E*_α_* were considered to be proportional to the errors in the slope of the linear regression using the KAS method for each α,^57^ and the error in *ln A*_*α*_ values was considered proportional to that in E*_α_*.

## Results and Discussion

### Short-Term Thermal Stability

**Figure 2:**
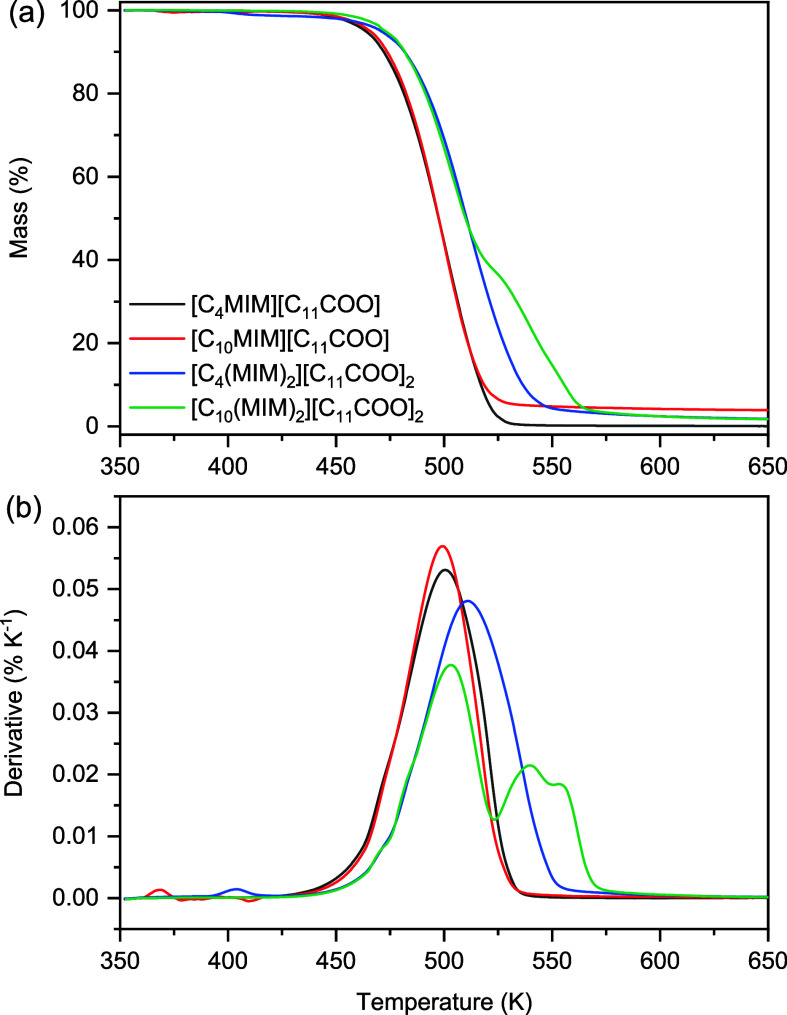
(a) Mass loss and (b) derivative curves of the studied ILs obtained at 10 K min^–1^.

The short-term thermal stability of the studied ILs was determined using a single-run TGA experiment with a heating rate of 10 K min^–1^. The results are shown in Figure 2 and encapsulated in Table 1, where T_0.05_ is the temperature at which 5% mass loss occurred and *T*_d_ is the temperature at the maximum decomposition rate (the maximum peak of the derivative curve). From Figure 2, it can be seen that the monocationic ILs presented very similar decomposition profiles, whereas for the dicationic ILs (DILs), there was variability in the shape and position of the curves in relation to the monocationic ILs, as well as between each DIL. Dicationic [C_10_(MIM)_2_][C_11_COO]_2_ was the only IL that presented a clear multistep decomposition process during the single-run TGA experiments. Furthermore, the DILs demonstrated slightly enhanced thermal stabilities relative to their monocationic analogs, as indicated by the T_0.05_ values in Table 1, which is a well-suited parameter as at 5% decomposition there are considerable quantities of decomposition products in the reaction medium, which is problematic for practical applications. Overall, the order of thermal stability for the ILs reported herein is [C_10_(MIM)_2_][C_11_COO]_2_ > [C_4_(MIM)_2_][C_11_COO]_2_ > [C_10_MIM][C_11_COO] ≈ [C_4_MIM][C_11_COO].

**Table 1:** Results from the Single-run TGA Experiments with a Heating Rate of 10 K min^–1^

IL	T_0.05_ (K)^a^	T_d_ (K)	Mass loss (%)^a^	Volatile content (%)^b^
[C_4_MIM][C_11_COO]	464	500	>99	7
[C_10_MIM][C_11_COO]	465	499	96	3
[C_4_(MIM)_2_][C_11_COO]_2_	470	511	98	12
[C_10_(MIM)_2_][C_11_COO]_2_	482	503, 540, 553	98	3

a 100% mass was considered as the mass after the elimination of volatiles.

b Difference in mass before and after the isothermal period prior to heating.

The ILs reported here were less thermally stable than their bromide analogs, with *T*_d_ values of 573,^58^ 575,^59^ 605,^60^ and 589 K^50^ for [C_4_MIM][Br], [C_10_MIM][Br], [C_4_(MIM)_2_][Br]_2_, and [C_10_(MIM)_2_][Br]_2_, respectively. This is a common trend for carboxylate-containing ILs and is associated with factors such as increased basicity of the anion and reduced crystallinity compared to bromide-containing ILs.^43,49,50,60^ Additionally, the ILs synthesized here were also less thermally stable compared to ILs that incorporate other common inorganic anions (e.g., BF_4_^–^, PF_6_^–^, NTf_2_^–^, etc.).^58^ The *T*_d_ values were used to evaluate thermal stability because few studies have reported the decomposition temperature of ILs at a fixed mass-loss percentage. However, in our previous work, we found that the value of T_0.05_ for [C_10_MIM][Br] was 502 K,^44^ which supports the trend of increased thermal stability for bromide-containing ILs.

### Kinetics of Thermal Decomposition

The short-term thermal stability determined using a single heating rate (Table 1) is known to overestimate the thermal stability of ILs.^61−63^ One strategy to overcome this limitation is to analyze the kinetic profile of thermal decomposition, preferably using an isoconversional methodology. In addition to information on the thermal stability of ILs, this type of analysis provides insight into the mechanism for thermal decomposition, and consequently, valuable information regarding the decomposition products, which may include undesirable, harmful compounds.^42^

**Figure 3:**
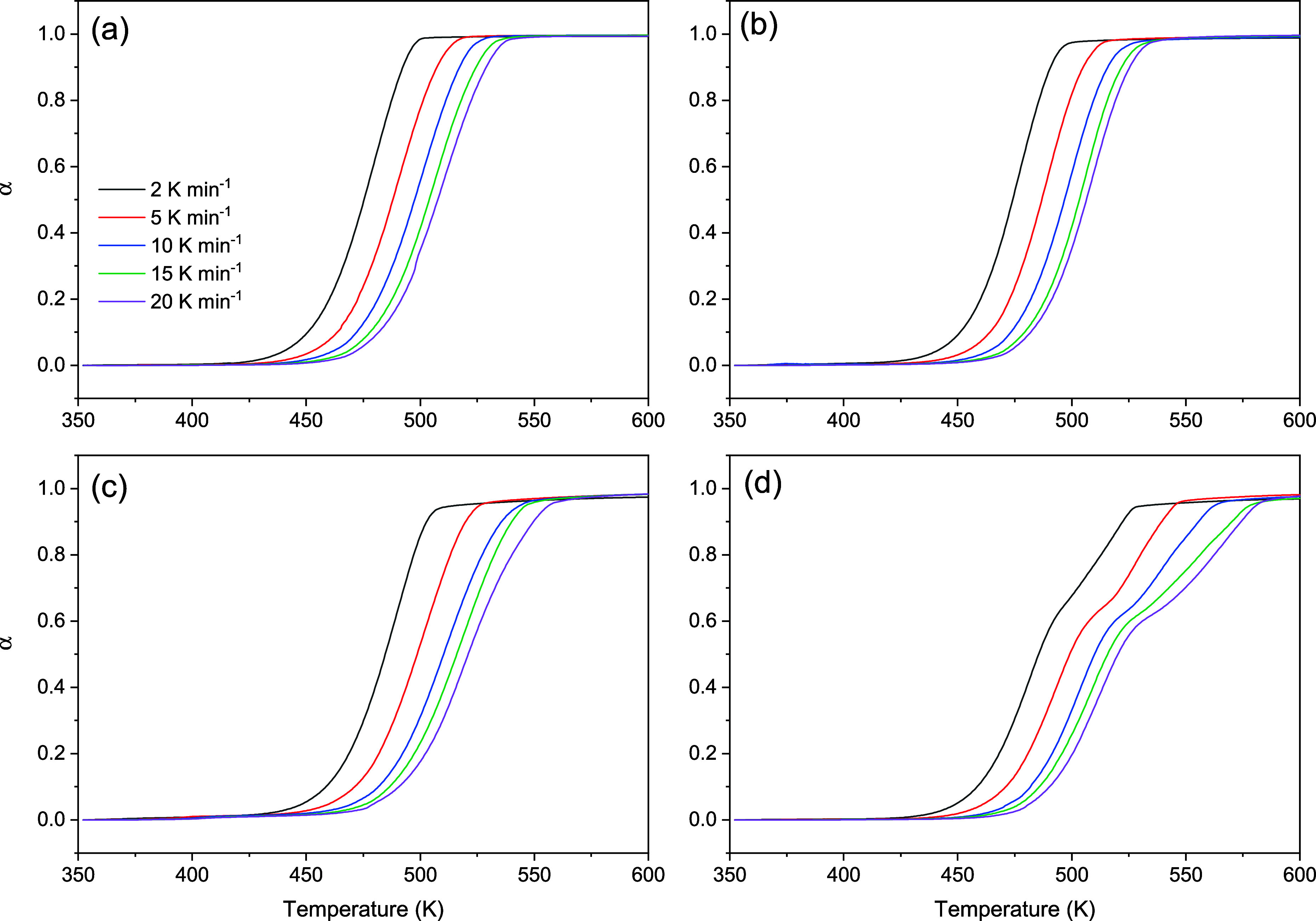
Curves of α vs temperature (K) at different heating rates for (a) [C_4_MIM][C_11_COO]; (b) [C_10_MIM][C_11_COO]; (c) [C_4_(MIM)_2_][C_11_COO]_2_ and (d) [C_10_(MIM)_2_][C_11_COO]_2_.

Kinetic analysis of the thermal decomposition of the ILs studied here was performed using the KAS isoconversional method. Samples of the ILs were heated at different heating rates, and the extent of conversion, α, was calculated as a function of temperature, as shown in Figure 3. As expected, upon increasing the heating rate, the decomposition curve shifted to higher temperatures. In addition, except for [C_10_(MIM)_2_][C_11_COO]_2_, the ILs exhibited seemingly single-step decomposition profiles at all heating rates. Although [C_10_(MIM)_2_][C_11_COO]_2_ exhibited a multistep curve profile, the steps were not sufficiently separated to be analyzed individually, so the whole curves were used for determining α. In the kinetic analysis, the same α value was obtained at different temperatures depending on the heating rate, which was used in the KAS eq 2 to determine E*_α_* at each chosen α.

**Figure 4:**
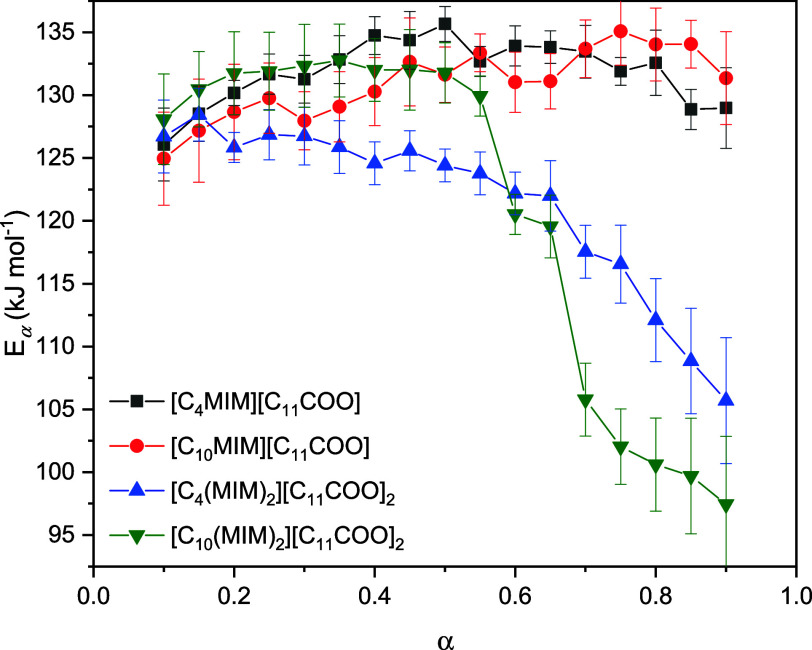
Variation in E*_α_* with α determined using the KAS isoconversional method.

The E*_α_* values were calculated at α = 0.1–0.9 in increments of 0.05, and the resulting curves are shown in Figure 4; individual values are summarized in Table S1. To determine the order of thermal stability from Figure 4, only the first half of the decomposition process was considered because as decomposition progresses, the amount of decomposition products increases and, consequently, the sample increasingly deviates from its original composition. As a result, it is more instructive to analyze the thermal stability of the ILs in the early stages of decomposition.

At the early stages of thermal decomposition (i.e., α = 0.1–0.3), the values of E*_α_* are not considerably different between each IL, as indicated by the error bars. This observation indicates that the main contributor to the thermal stability during the beginning stages of decomposition is the dodecanoate anion, as different cations did not considerably affect E*_α_*. In addition, the results of the kinetic analysis revealed that a difference of less than 20 K in the T_0.05_ values for the mono- and dicationic ILs (Table 1) was not as relevant as the short-term thermal stability analysis may imply. Therefore, these results indicate that when considering the application of these IL, other properties aside from thermal stability must be considered as there is little difference between ILs.

The profiles of the isoconversional curves provide additional information regarding the decomposition mechanism. If there is a considerable variation in E*_α_* with α (considered here as higher than 10% of the mean E*_α_* value), then the decomposition process is comprised of more than one reaction contributing to the overall E*_α_* value.^42^ The monocationic ILs did not show considerable variation in E*_α_* with α and, therefore, undergo thermal decomposition by a single step. However, in the case of the DILs, both showed variable E*_α_*, indicating multiple reactions taking place during the thermal decomposition process. It is worth noting that the values of isoconversional activation energies calculated for the DILs using the KAS method may deviate from the true activation energy of the process, since this method is based on a single-step approximation. However, this deviation is compensated by information obtained about the decomposition mechanism. In the case of the studied ILs, it can be clearly seen that the second cationic head on the cation dramatically affects the decomposition mechanism, despite the fact that the E*_α_* values at the beginning of thermal decomposition are similar to those of monocationic ILs.

**Figure 5:**
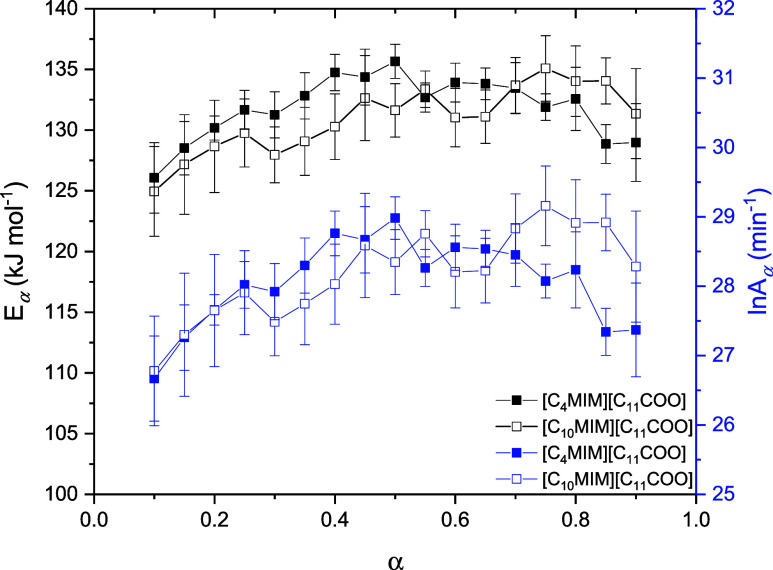
Variation in E*_α_* and *ln A*_*α*_ with α for the thermal decomposition process of the studied monocationic ILs.

Since [C_4_MIM][C_11_COO] and [C_10_MIM][C_11_COO] exhibited single-step decomposition profiles, the pre-exponential factor (*A*_*α*_) and the kinetic model (*f(α)*) curves were determined for these ILs. To calculate *A*_*α*_ using the compensation effect, the α vs *T* curves obtained at 10 K min^–1^ were applied to the same single-step kinetic equation and theoretical kinetic models as in our previous works.^43,44^ The set of E*_αj_* and *ln A*_*αj*_ values obtained for both ILs showed a good linear correlation (see plots in Figures S13 and S14 and the values in Table S2) according to eq 3. Having calculated the values of *a* and *b* in eq 3 for both ILs, the E*_α_* values obtained using the KAS isoconversional method were used to calculate *ln A*_*α*_ for each α considered, as is shown in Figure 5 (numerical values of *ln A*_*α*_ are provided in Table S3).

As expected, the *ln A*_*α*_ curves exhibit a profile similar to that of the E*_α_* curves. The mean values of E*_α_* (132 ± 3 kJ mol^–1^ for [C_4_MIM][C_11_COO] and 131 ± 3 kJ mol^–1^ for [C_10_MIM][C_11_COO]) and *ln A*_*α*_ (28 ± 1 min^–1^ for [C_4_MIM][C_11_COO] and 28 ± 1 min^–1^ for [C_10_MIM][C_11_COO]) are very similar for both ILs, supporting their indistinguishable thermal stabilities. The similarity in *ln A*_*α*_ values indicates that approximately the same number of collisions occur during thermal decomposition of both ILs. Moreover, the similar energy barrier required for decomposition (E*_α_*) facilitates the same number of successful reactions and, therefore, the same thermal stability. Determining the *ln A*_*α*_ profile for the dicationic ILs was not possible using the compensation effect, but a similar correlation between E*_α_* and *ln A*_*α*_ is to be expected. However, this investigation is beyond the scope of the present work.

Compared to previously reported kinetic parameters (i.e., E*_α_* and *ln A*_*α*_) and variations in α for carboxylate-containing ILs, [C_10_MIM][C_11_COO] shows similar values and variation profiles to [C_10_MIM][C_3_COO], with both demonstrating decreased thermal stability than their corresponding bromide-containing IL precursor, [C_10_MIM][Br].^44^ The profile for the variation in E*_α_* relative to α for both carboxylate-containing ILs shows higher values of E*_α_* than for [C_10_MIM][Br]. However, they also exhibit higher values of *ln A*_*α*_, indicating that an increase in the energy barrier is accompanied by an increase in collision frequency, leading to reduced thermal stability as a result of an increase in productive decomposition reactions.^44^ The similarity in thermal stability between [C_10_MIM][C_11_COO] and [C_10_MIM][C_3_COO] can be attributed to similar decomposition mechanisms that depend only on the presence of the carboxylate group and not on the size of the anion. The decomposition mechanism for [C_10_MIM][C_11_COO] is discussed in the following section.

**Figure 6:**
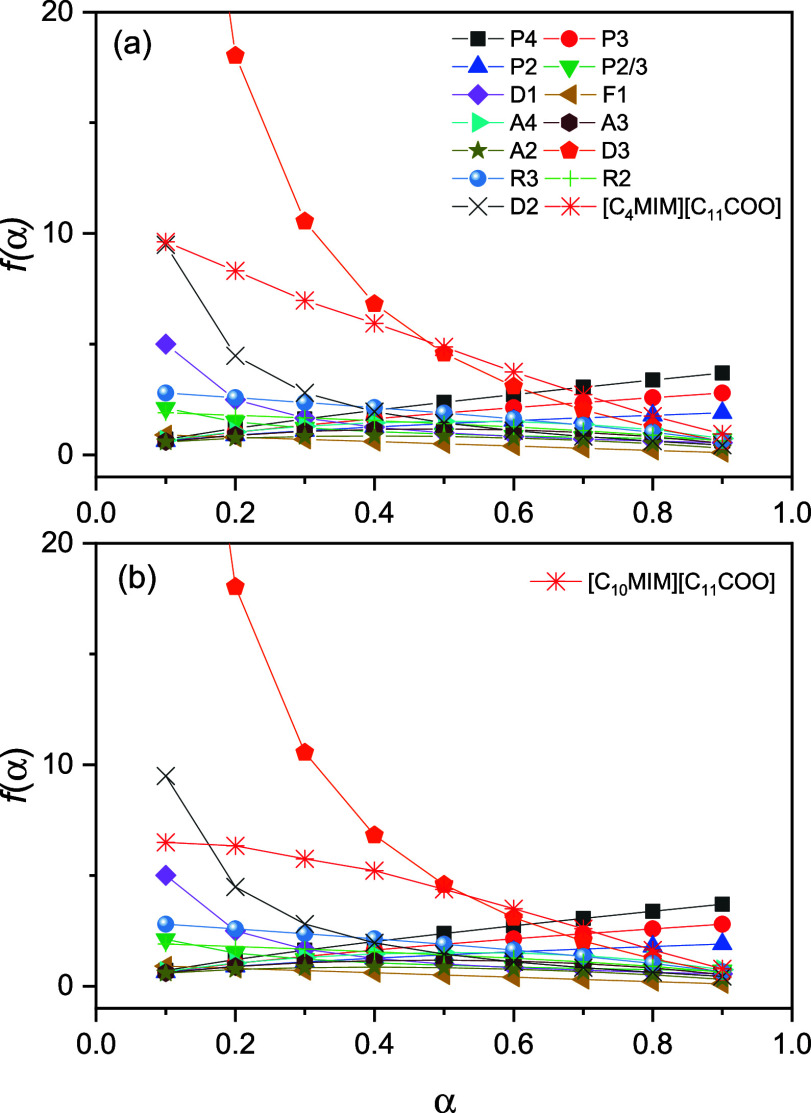
*f(α)* curves for (a) [C_4_MIM][C_11_COO] and (b) [C_10_MIM][C_11_COO].

Following the determination of *ln A*_*α*_, eq 5 was used to calculate *f(α)* for α = 0.1–0.9 (in increments of 0.1), and the curve profile was compared to a set of commonly used theoretical kinetic models, as shown in Figure 6. According to Khawam and Flanagam,^64^ theoretical kinetic models primarily consider thermal decomposition of crystalline solids. In the case of the monocationic ILs, the *f(α)* curves did not match any of the kinetic models, so no additional information regarding the mechanism for thermal decomposition could be obtained using this method. However, we hope that these results can be used in future investigations of the thermal stability of carboxylate-containing ILs.

### Mechanism of Thermal Decomposition

**Scheme 1:**
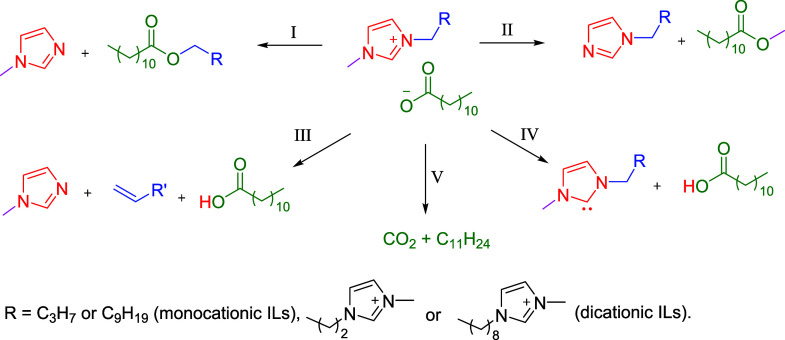
Thermal Decomposition Pathways for the Synthesized ILs One of the anions in each of the DILs was omitted to simplify the reaction scheme. R′ in mechanism III differs from R by only one methylene group.

Alkylimidazolium-based ILs are known to decompose via one or more of the following reaction mechanisms: (I) Nucleophilic substitution at the side chain methylene group; (II) nucleophilic substitution at the methyl group of the methylimidazolium ring; (III) an elimination reaction at the side chain; or (IV) formation of *N*-heterocyclic carbenes (NHC) (Scheme 1).^43,47−49,65,66^ For ILs containing carboxylate anions, the formation of carbon dioxide (mechanism (V)) is also possible (Scheme 1).

**Figure 7:**
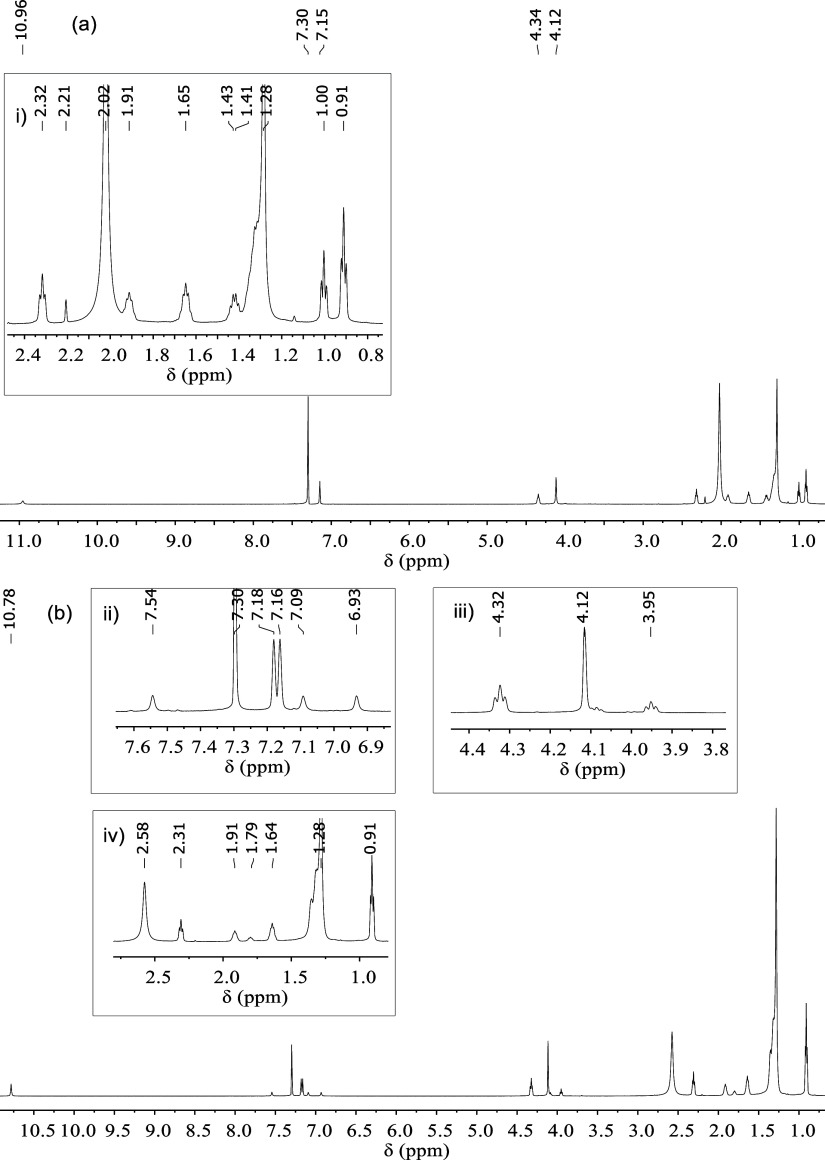
^1^H NMR spectra of the decomposition residues of (a) [C_4_MIM][C_11_COO] and (b) [C_10_MIM][C_11_COO] (CDCl_3_, 600 MHz, 298 K).

The mechanism for thermal decomposition of the ILs was also investigated by analyzing the residue remaining following thermal decomposition by ^1^H NMR spectroscopy; samples were heated to 493 K, and the remaining residue following cooling was dissolved in CDCl_3_. The presence of additional resonances in the NMR spectra indicates there are decomposition products in the samples. The ^1^H NMR spectra for [C_4_MIM][C_11_COO] and [C_10_MIM][C_11_COO] are shown in Figure 7, and it can be seen that [C_4_MIM][C_11_COO] (Figure 7a) gives rise to additional resonances in the 2.4–0.8 ppm region (i.e., 2.32, 1.91, and 1.65 ppm) while only one set of imidazolium resonances represent those associated with the IL. The absence of new peaks in the aromatic region is suggestive of the formation of a more volatile imidazole-containing species, such as methylimidazole, following decomposition mechanisms I and III (Scheme 1), compared to butylimidazole formed in mechanism II. The new signals in the spectrum could also be attributed to esters formed during the decomposition process, although the proton resonances closest to the −O–C(=O)– moiety, which are expected to appear in the 4.0–3.0 ppm region,^67^ are absent. Therefore, the results of ^1^H NMR analysis of the decomposition residue of [C_4_MIM][C_11_COO] were not sufficient to determine the reaction mechanisms involved in the thermal decomposition of this IL.

However, in the case of [C_10_MIM][C_11_COO] (Figure 7b), additional signals were observed over the entire spectrum; there was a set of resonances of similar intensity at 7.54, 7.09, and 6.93 ppm (expansion (ii) in Figure 7b) indicating the formation of neutral imidazole species, as a loss of cationic charge would result in a shift of the resonances to lower δ_H_. This observation points to decomposition mechanisms I–III as possible reaction pathways for this IL. The remaining residue from the thermal decomposition of [C_10_MIM][C_11_COO] gave rise to a new resonance at 3.95 ppm (expansion (iii) in Figure 7b) that can be assigned to the protons closest to the ester moiety, narrowing the possible reaction mechanisms to I and II. Considering the observation of resonances associated with neutral imidazole, mechanism II seems most likely as the imidazole species formed is less volatile. The new signals in the 3.0–0.5 ppm region can be assigned to the methylene group of both ester and imidazole species formed during decomposition. The similarities in thermal stability (E*_a_* and *ln A* versus α profiles) between [C_10_MIM][C_11_COO] and [C_10_MIM][C_3_COO], as was highlighted in the discussion pertaining to isoconversional thermal stability, can be attributed to decomposition mechanism II being the most probable degradation pathway for both ILs, as indicated here and in our previous work.^44^

**Figure 8:**
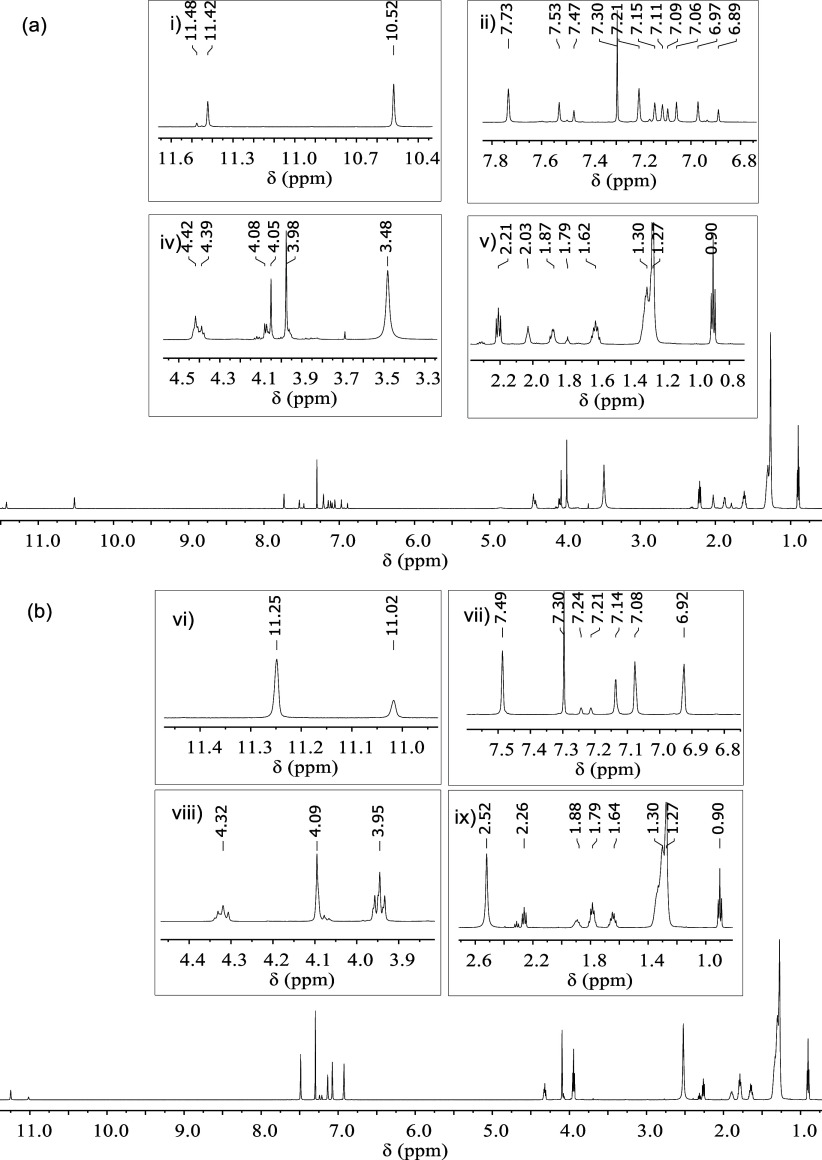
^1^H NMR spectra of the decomposition residue of (a) [C_4_(MIM)_2_][C_11_COO]_2_ and (b) [C_10_(MIM)_2_][C_11_COO]_2_ (CDCl_3_, 600 MHz, 298 K).

The ^1^H NMR spectra of the residue from the thermal decomposition of the DILs are shown in Figure 8. For [C_4_(MIM)_2_][C_11_COO]_2_ (Figure 8a), several additional peaks are indicative of decomposition products. The various new signals in the imidazole ring region indicate the formation of more than one imidazole-containing species as a decomposition product, including at least one positively charged structure, as there are extra peaks above 10 ppm (expansion (i) in Figure 8a), which are characteristic of the proton located at position 2 (*N* = C**H**-N) of the charged imidazole ring. All mechanisms I–IV in Scheme 1 involve the formation of positively charged products during the thermal decomposition of the DILs. There are also signals at 7.73, 7.53, and 7.47 ppm (expansion (ii) in Figure 8a) that can be assigned neutral imidazole species. The signals between 7.21 and 6.89 ppm correspond to the protons at positions 4 and 5 (C**H**=C**H**) of the imidazole ring in all of these degradation products. Moreover, there are resonances located at 3.98 and 3.48 ppm that could be assigned to the methylene hydrogen atoms closest to the ester group, indicative of the formation of esters and hinting to mechanisms I and II being operative during the decomposition process (inset (iv) of Figure 8a).

Analysis of the ^1^H NMR spectrum of the decomposition residue of [C_10_(MIM)_2_][C_11_COO]_2_ (Figure 8b) reveals a profile similar to that of [C_4_(MIM)_2_][C_11_COO]_2_. There is a potentially positively charged decomposition product, giving rise to a resonance above 10 ppm (expansion (vi) in Figure 8b). The signal at 7.49 ppm (expansion (vii) in Figure 8b) indicates the presence of neutral imidazole species in the sample. For these DILs, the signal at 3.95 ppm (expansion (viii) in Figure 8b) indicates the presence of ester decomposition products, consistent with mechanisms I and II.

**Figure 9:**
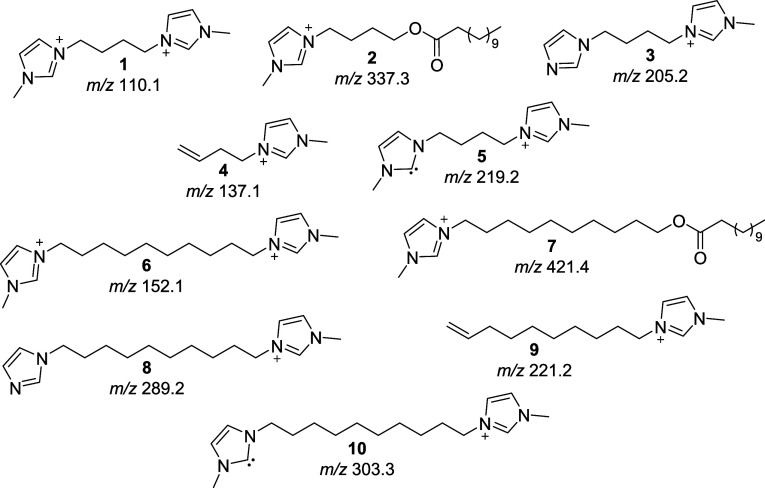
Molecular structures of possible decomposition products from the DILs and their respective predicted *m*/*z*.

From the ^1^H NMR spectra in Figure 8, it is clear that one of the decomposition products is a positively charged imidazole species. Decomposition mechanisms I–IV involve the formation of such structures, and these products can be differentiated based on their mass-to-charge ratios (*m*/*z*); Figure 9 shows the predicted *m*/*z* values for various cations formed during the thermal decomposition of the DILs. Structures **1** and **6** correspond to the unreacted cations, **2** and **7** to the products in mechanism I, **3** and **8** are formed in mechanism II, **4** and **9** are from mechanism III, and **5** and **10** are formed in mechanism IV.

**Figure 10:**
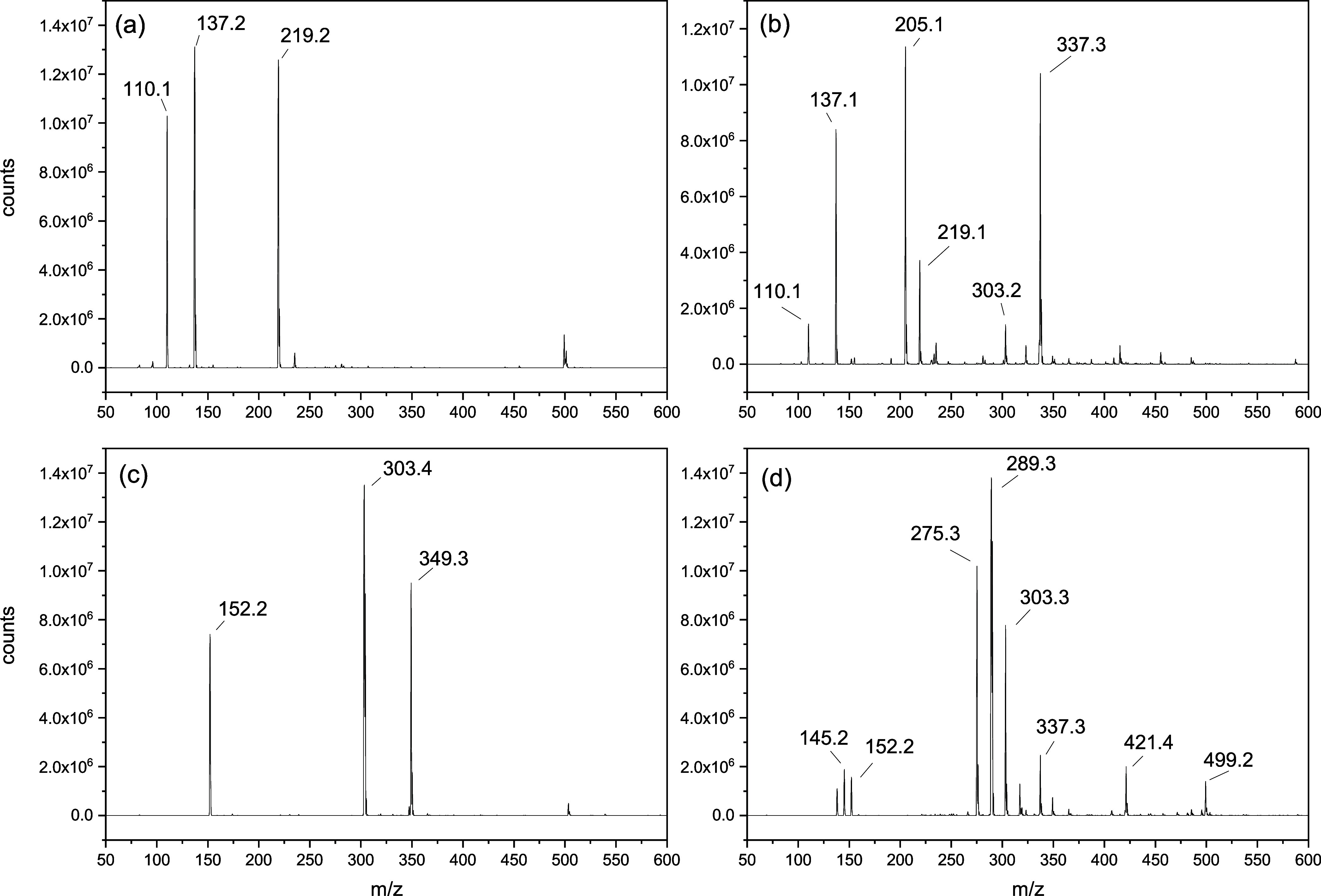
ESI-MS spectra in positive ion mode of (a) [C_4_(MIM)_2_][C_11_COO]_2_ and (b) [C_10_(MIM)_2_][C_11_COO]_2_, and of the remaining residues from thermal decomposition of (c) [C_4_(MIM)_2_][C_11_COO]_2_ and (d) [C_10_(MIM)_2_][C_11_COO]_2_.

Therefore, the remaining residue from the thermal decomposition of the DILs was analyzed by ESI-MS, and the resultant mass spectra are shown in Figure 10 along with the mass spectra of the pure DILs. Aside from the signal related to the dication, there are two additional peaks in the mass spectra of the pure DILs. These peaks are found at *m*/*z* 137.2 and 219.2 for [C_4_(MIM)_2_][C_11_COO]_2_ (Figure 10a) and *m*/*z* 303.4 and 349.3 for [C_10_(MIM)_2_][C_11_COO]_2_ (Figure 10c) and may originate from the electrospraying process. Except for *m*/*z* 349.3, these additional signals possess the same *m*/*z* as decomposition products **4**, **5**, and **10**, respectively, corresponding to the decomposition products in mechanisms III and IV for [C_4_(MIM)_2_][C_11_COO]_2_ and IV for [C_10_(MIM)_2_][C_11_COO]_2_. Consequently, if these are the mechanisms involved in the thermal decomposition of the DILs, they cannot be differentiated because the signals of the decomposition products would also be found in the spectra of the pure DILs.

The mass spectrum of the residue from the thermal decomposition of [C_4_(MIM)_2_][C_11_COO]_2_ in Figure 10b shows peaks at *m*/*z* 205.1 and 337.3, corresponding to decomposition products **3** and **2**, formed in mechanisms II and I, respectively. These signals have similar intensities, indicating that there is no preference between the two mechanisms for the thermal decomposition of the DIL. In the case of [C_10_(MIM)_2_][C_11_COO]_2_, the mass spectrum, shown in Figure 10d, contains peaks from the decomposition products **7** and **8** formed in mechanisms I and II at *m*/*z* 421.4 and 289.3, respectively. For this DIL, there was a significant difference in the intensities of these signals, with the decomposition product **8** being much more abundant than **7**, indicating the predominance of mechanism II. These results are similar to those obtained for DILs [C_4_(MIM)_2_][C_3_COO]_2_, [C_4_(MIM)_2_][C_6_COO]_2_, and [C_10_(MIM)_2_][C_3_COO]_2_, indicating a complex thermal decomposition process with mechanisms I and II as the primary decomposition pathways.^43^

In the mass spectrum of the residues remaining following degradation of [C_10_(MIM)_2_][C_11_COO]_2_ (Figure 10d), there is an intense signal at *m*/*z* 275.3 that does not correspond to any of the expected decomposition products shown in Figure 9, indicating a decomposition reaction that is not one of the main pathways reported in the literature. We attempted to determine the identity of this decomposition product and how it was produced but were unsuccessful. In addition, there were other new peaks at *m*/*z* 145.2, 337.3, and 499.2, albeit less intense, which did not correspond to the decomposition products shown in Figure 9. It is well-known in the literature that imidazolium-based ILs, unsubstituted in the 2-position, can undergo deprotonation following mechanism IV and rapidly rearrange the highly reactive NHC species formed.^47−49^ Therefore, it can be the case that the unidentified products in the ESI-MS spectra are formed from these NHC species that indiscriminately react with surrounding molecules during electrospraying. These observations indicate that [C_10_(MIM)_2_][C_11_COO]_2_ decomposes via a complex thermally stimulated process, although mechanism II can still be considered the primary pathway.

A correlation between the variation in E*_a_* with α for the DILs, shown in Figure 4, can be made with the degradation analysis performed to elucidate the decomposition mechanism. In Figure 4, both DILs showed a considerable variation in E*_a_* with α, which can be attributed to more than one reaction contributing to the overall E*_a_* during thermal decomposition. From the analysis of the decomposition products and mechanism, it is clear that the DILs decompose via more than one decomposition pathway, thus leading to the observed variation in E*_a_* with α during the isoconversional analysis.

**Figure 11:**
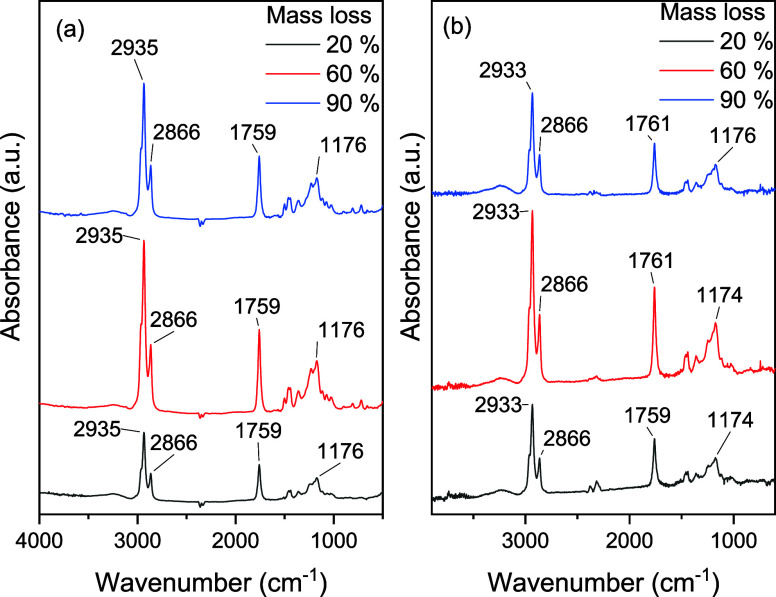
FTIR spectra of the gas flow from the TGA furnace for (a) [C_4_MIM][C_11_COO] and (b) [C_10_MIM][C_11_COO].

In the case of the monocationic ILs [C_4_MIM][C_11_COO] and [C_10_MIM][C_11_COO], the thermal decomposition mechanisms were not investigated using ESI-MS because the decomposition products formed, according to Scheme 1, are not charged. Instead, gas flow from the TGA furnace was applied to the FTIR detector. FTIR spectra were acquired for both ILs at varying decomposition percentages (20%, 60%, and 90% mass loss) and are shown in Figure 11. The spectra showed little variation in the main bands at different mass loss values when comparing the two ILs. The bands in the 2950–2850 cm^–1^ region can be assigned to axial deformation of the C–H bonds present in the decomposition products in all mechanisms shown in Scheme 1, and the bands at approximately 1760 cm^–1^ can potentially be assigned to the formation of carboxylic acids. However, the characteristic broad O–H stretching band in the 3300–2500 cm^–1^ region was not observed. Furthermore, the C=O stretching vibration was slightly above the 1750–1735 cm^–1^ region expected for esters; however a band at approximately 1175 cm^–1^ corresponding to the ester C–O stretching vibration confirmed the presence of esters in the samples.^67^ As a result, the TGA-FTIR spectra indicate that the thermal decomposition of [C_4_MIM][C_11_COO] and [C_10_MIM][C_11_COO] occurs via mechanisms I or II, but their elucidation was not possible using this technique as both mechanisms may be simultaneously operative and therefore indistinguishable.

## Conclusions

In this work, two monocationic ILs ([C_4_MIM][C_11_COO] and [C_10_MIM][C_11_COO]) and two dicationic ILs ([C_4_(MIM)_2_][C_11_COO]_2_ and [C_10_(MIM)_2_][C_11_COO]_2_) were synthesized, structurally characterized, and their thermal stability studied by short-term TGA analysis and isoconversional kinetic analysis. The results of the isoconversional analysis showed that there were no significant differences in the thermal stabilities of the ILs, with all the compounds being thermally stable up to 450 K. In comparison with the literature, it was observed that an increase in the anion alkyl chain length did not necessarily improve the thermal stability of the monocationic ILs; therefore, when considering applications that depend on thermal stability, such as their use as lubricants, the choice of the IL carboxylate anion can be made based on the other physicochemical properties. In the case of the dicationic ILs, they presented a variable isoconversional activation energy and therefore access to their thermal stability profile was limited. Consequently, the use of more sophisticated isoconversional methodologies for these dicationic ILs is an interesting topic for future investigations. An analysis of the thermal decomposition residues using ^1^H NMR, ESI-MS, and TGA-FTIR showed that the main decomposition pathways were nucleophilic substitution at the lateral or spacer chains and at the methyl groups. For [C_4_(MIM)_2_][C_11_COO]_2_, both occurred to similar extents, as indicated by ESI-MS. For [C_10_(MIM)_2_][C_11_COO]_2_, the reaction at the methyl group prevailed, but other unidentified reactions occurred during the decomposition process. For the monocationic ILs, both mechanisms appear to occur simultaneously, as highlighted by the TGA-FTIR results. In addition, in the case of [C_10_MIM][C_11_COO], the ^1^H NMR analysis indicated that the reaction at the methyl group was the most likely reaction pathway.

## Supporting Information Available

NMR spectra of the ILs (Figures S1–S8), ATR-FTIR spectra of the ILs (Figures S9–S12), compensation effect plots for [C_4_MIM][C_11_COO] (Figure S13) and [C_10_MIM][C_11_COO] (Figure S14),E*_α_* and *ln A*_*α*_values (Tables S1 and S3), and E*_αj_* and *ln A*_*αj*_ values (Table S2)(PDF)
