# Supplementary material for: Effect of the Dodecanoate Anion on Thermal Stability and Decomposition Mechanism of Mono- and Dicationic Ionic Liquids
Source: ACS Omega. 2025 Feb 26;10(9):9514–26. doi: 10.1021/acsomega.4c10596 (PMC11904690; doi:10.1021/acsomega.4c10596)
Supplement: Supplementary file 1 — ao4c10596_si_001.pdf [file ao4c10596_si_001.pdf]

Effect of dodecanoate anion on thermal stability and decomposition mechanism of mono- and dicationic ionic liquids

Jean C. B. Vieira[1]*, Marcos A. Villetti[2], Caroline R. Bender[1] and Clarissa P. Frizzo[1]

[1]*NUQUIMHE, Department of Chemistry, Federal University of Santa Maria, Santa Maria, Brazil, 97105-900*

[2] *LEPOL, Department of Physics, Federal University of Santa Maria, Santa Maria, Brazil, 97105-900*

**e-mail:* jeanbauer96@gmail.com

SUPPORTING INFORMATION FILE

NMR spectra of the studied ILs 2

ATR-FTIR spectra of the studied ILs 6

Kinetic Analysis 7

NMR spectra of the studied ILs

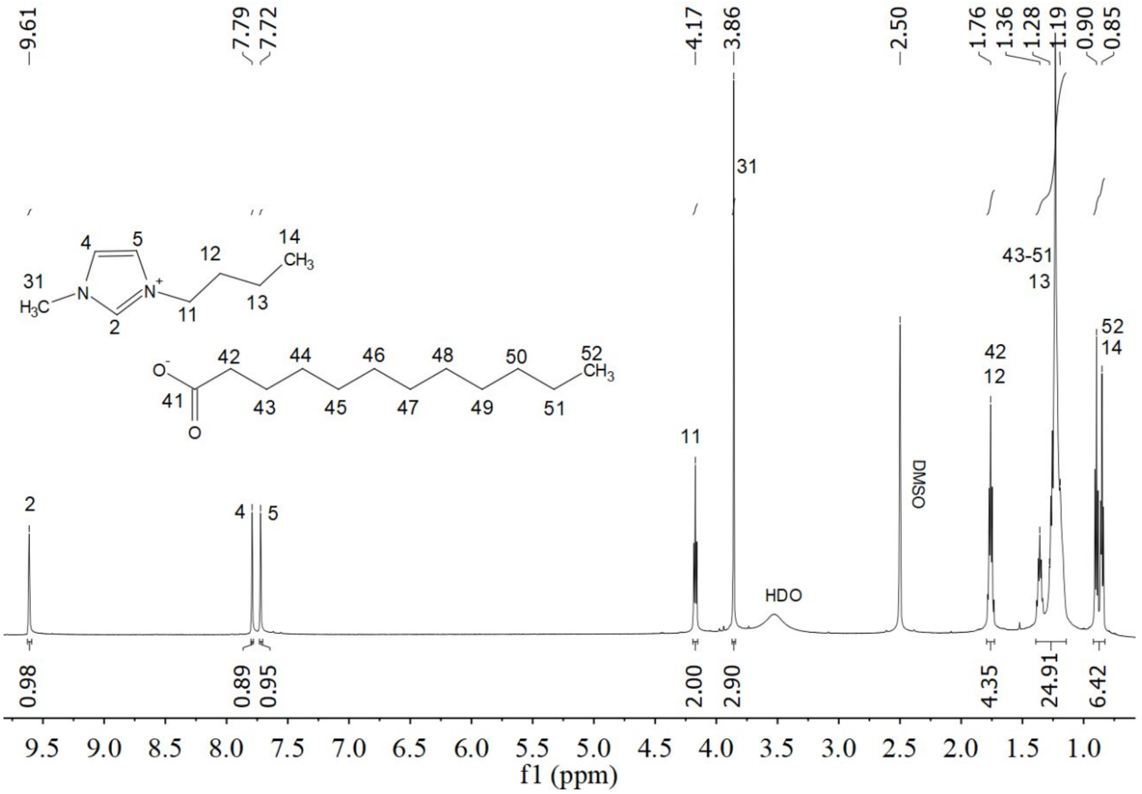

Figure S1. ^{1}H NMR of the IL [C_4MIM][C_{11}COO] (DMSO-d_6, 25 °C, 600 MHz).

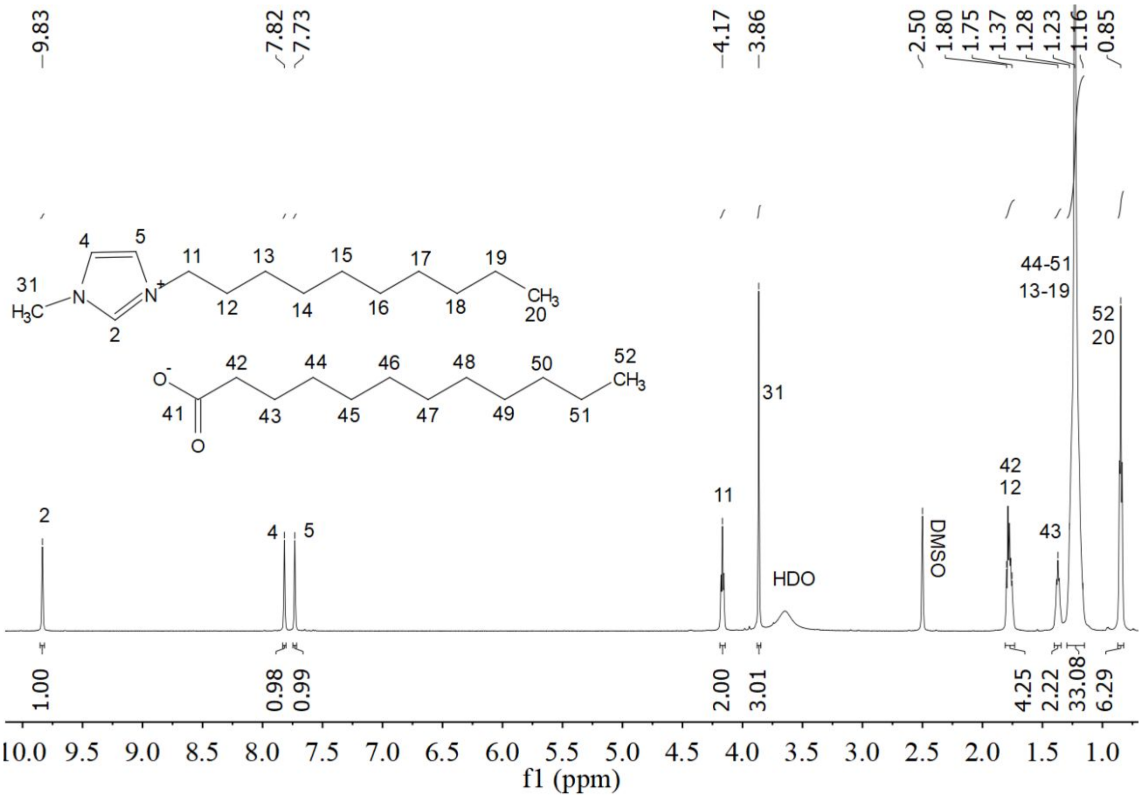

Figure S2. ^{1}H NMR of the IL [C_{10}MIM][C_{11}COO] (DMSO-d_6, 25 °C, 600 MHz).

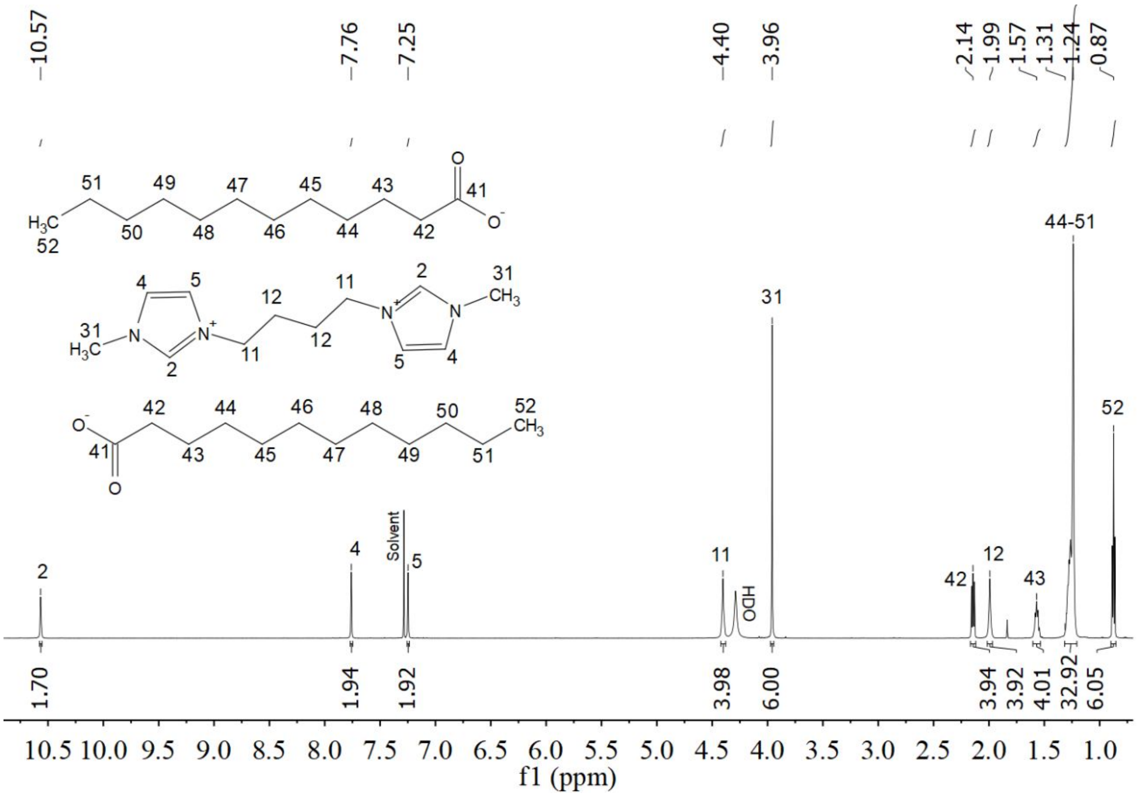

Figure S3. ^{1}H NMR of the IL $[C_4(MIM)_2][C_{11}COO]_2$ (DMSO-d_6, 25 °C, 600 MHz).

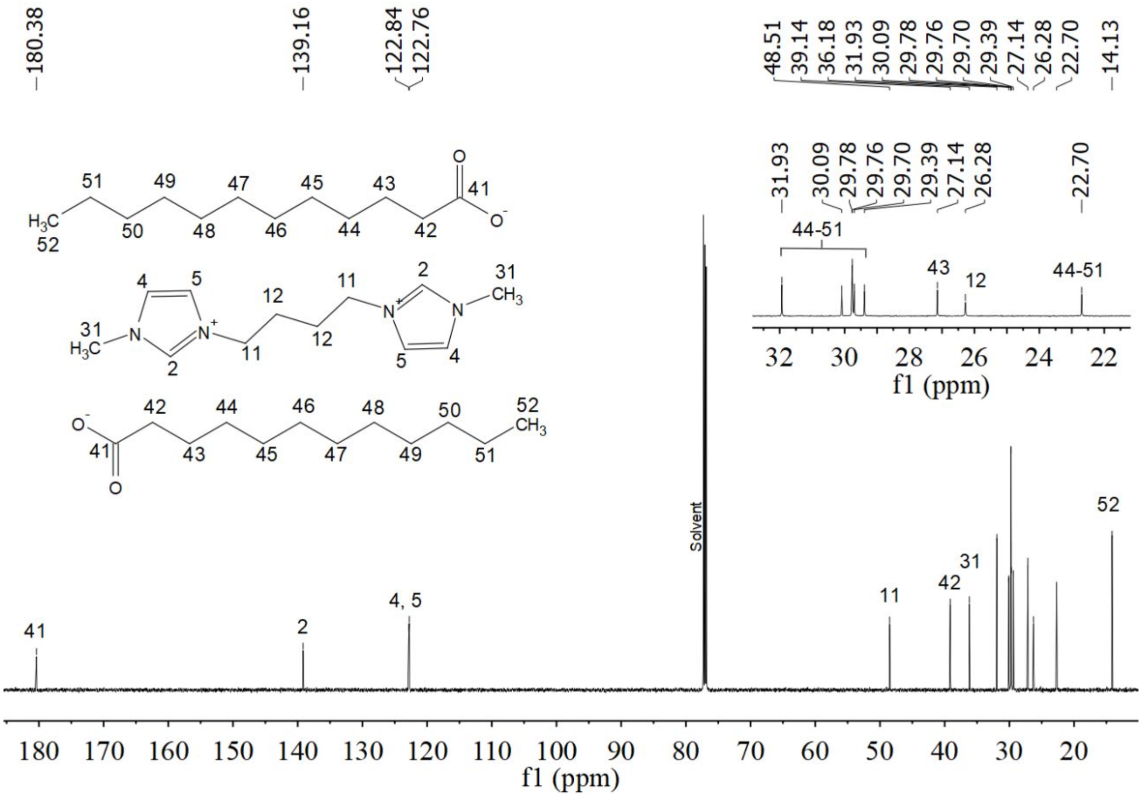

Figure S4. ^{13}C NMR of the IL $[C_4(MIM)_2][C_{11}COO]_2$ (DMSO-d_6, 25 °C, 150 MHz).

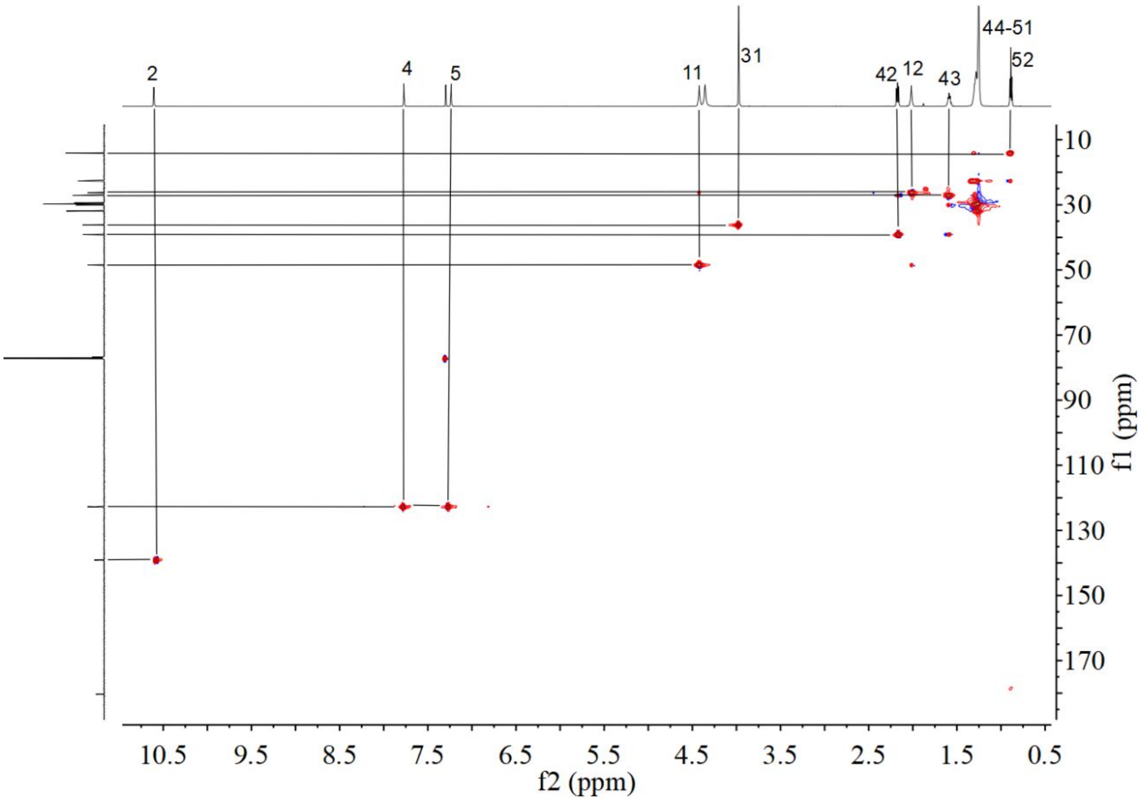

Figure S5. HSQC ^{1}H-^{13}C NMR spectrum of the IL $[C_4(MIM)_2][C_{11}COO]_2$ (DMSO-d_6, 25 °C, ^{1}H: 600 MHz, ^{13}C: 150 MHz).

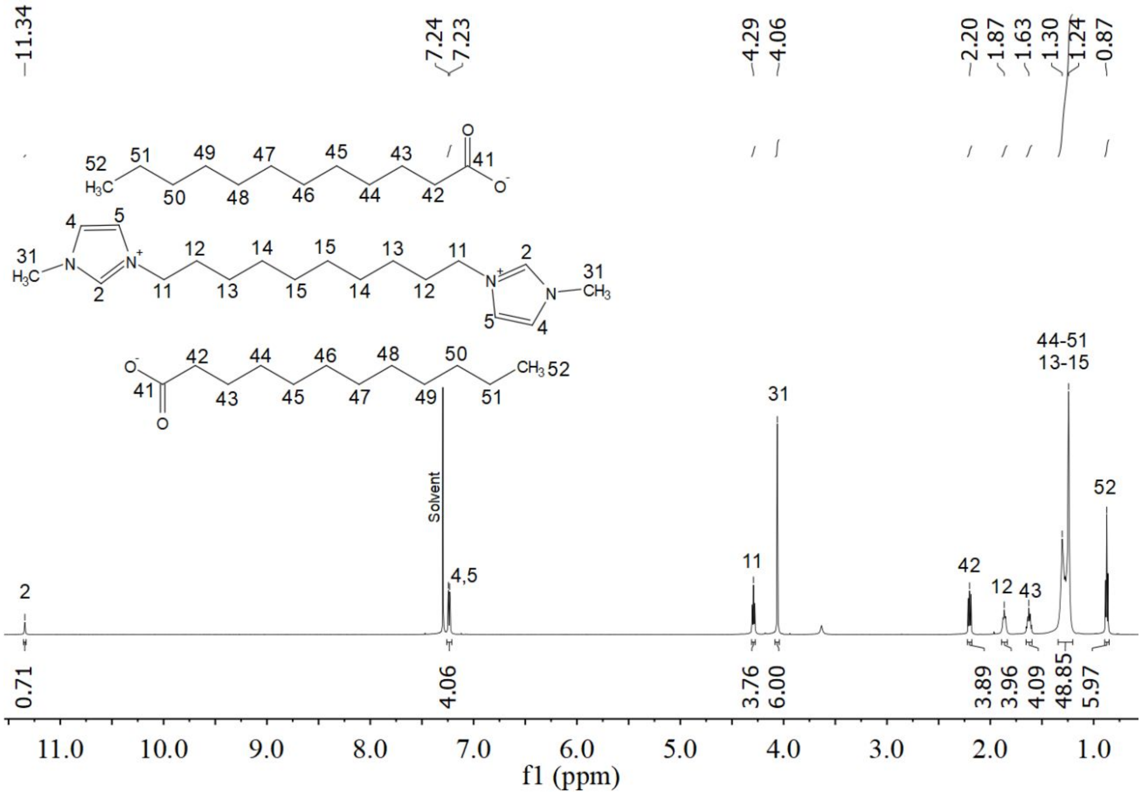

Figure S6. ^{1}H NMR of the IL $[C_{10}(MIM)_2][C_{11}COO]_2$ (DMSO-d_6, 25 °C, 600 MHz).

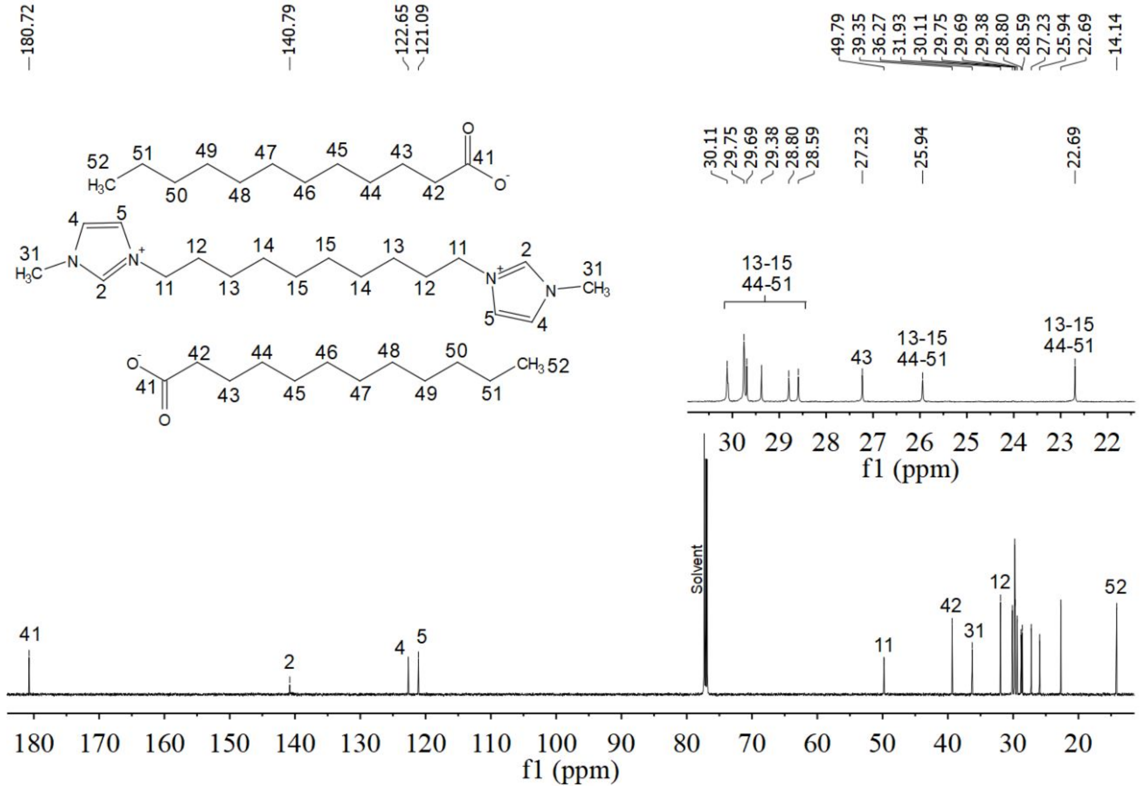

Figure S7. ^{13}C NMR of the IL $[C_{10}(MIM)_2][C_{11}COO]_2$ (DMSO-d_6, 25 °C, 150 MHz).

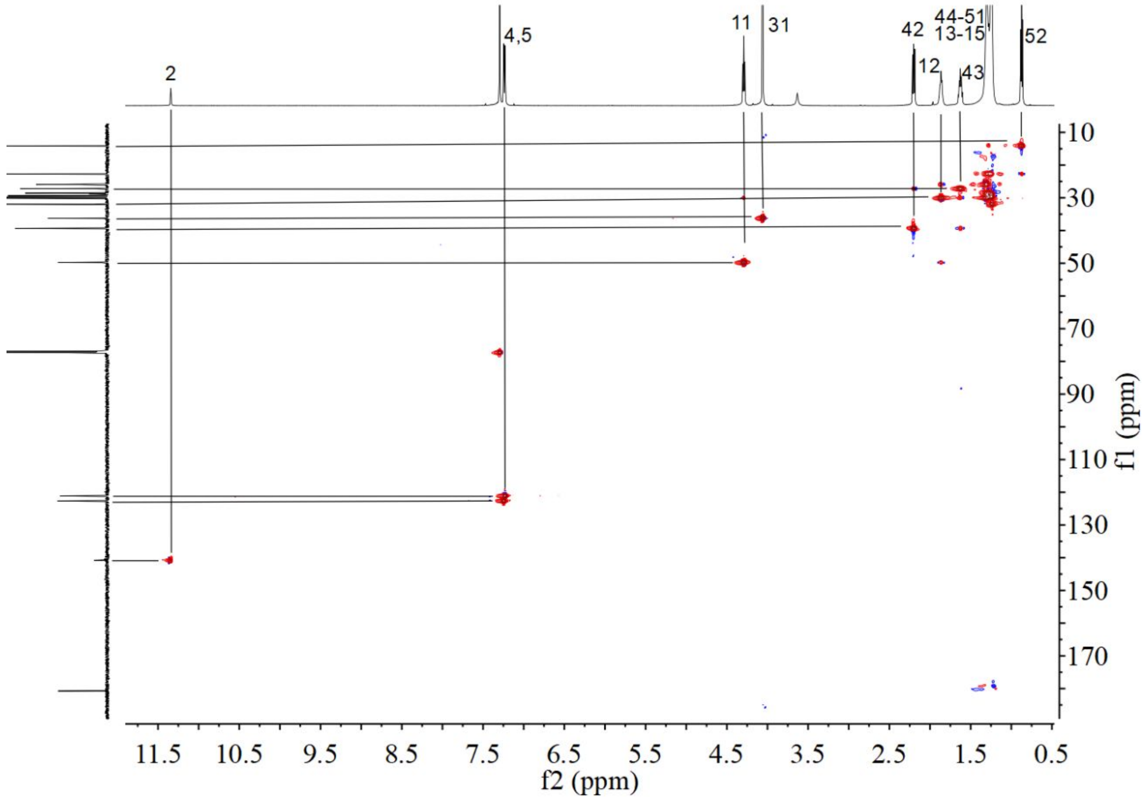

Figure S8. HSQC ^{1}H-^{13}C NMR spectrum of the IL $[C_{10}(MIM)_2][C_{11}COO]_2$ (DMSO-d_6, 25 °C, ^{1}H: 600 MHz, ^{13}C: 150 MHz).

ATR-FTIR spectra of the studied ILs

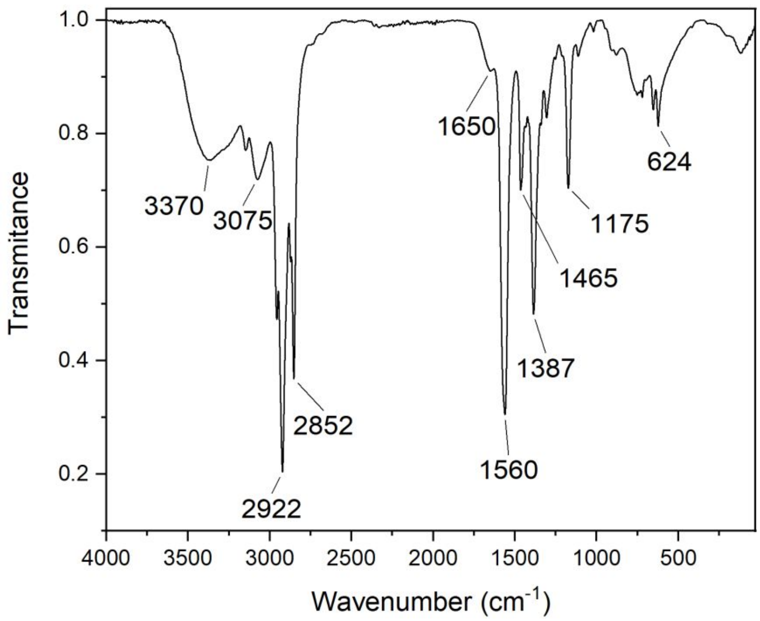

Figure S9. ATR-FTIR spectrum of the IL [C_4MIM][C_{11}COO].

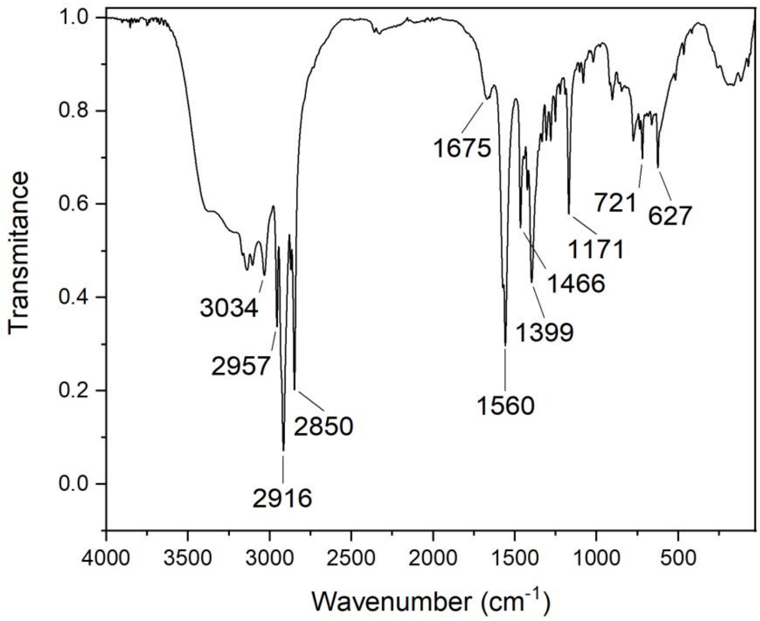

Figure S10. ATR-FTIR spectrum of the IL [C_{10}MIM][C_{11}COO].

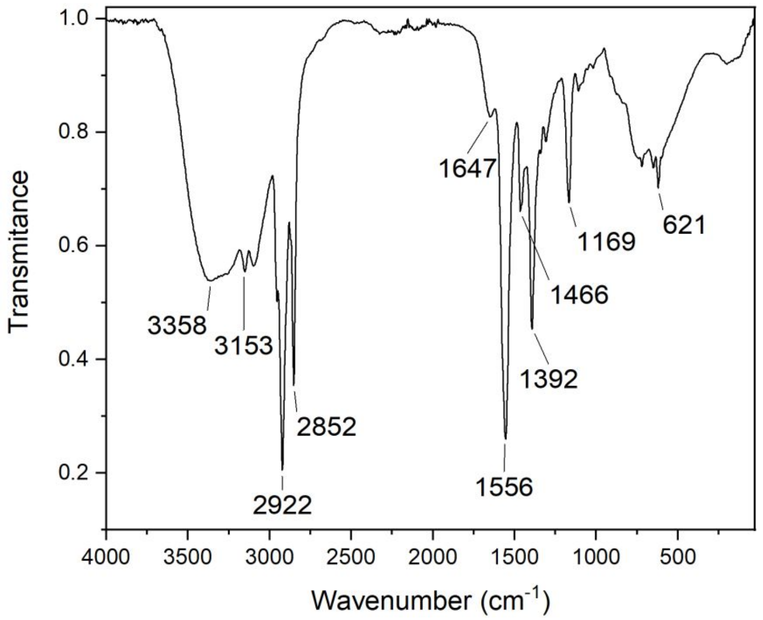

Figure S11. ATR-FTIR spectrum of the IL $[C_4(MIM)_2][C_{11}COO]_2$.

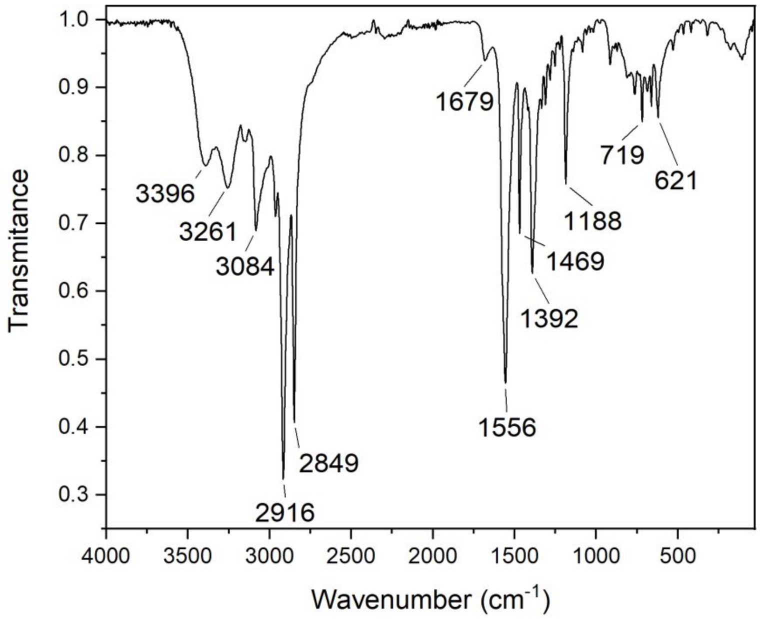

Figure S12. ATR-FTIR spectrum of the IL $[C_{10}(MIM)_2][C_{11}COO]_2$.

Kinetic Analysis

Table S1. Values of E_α (kJ mol^{-1}) for the studied ILs determined using the KAS method.

α	$[C_4MIM][C_{11}COO]$		$[C_{10}MIM][C_{11}COO]$		$[C_4(MIM)_2][C_{11}COO]_2$		$[C_{10}(MIM)_2][C_{11}COO]_2$	
	E_α (kJ mol^{-1})	Error (kJ mol^{-1})	E_α (kJ mol^{-1})	Error (kJ mol^{-1})	E_α (kJ mol^{-1})	Error (kJ mol^{-1})	E_α (kJ mol^{-1})	Error (kJ mol^{-1})
0.1	126.06	2.9	124.95	3.7	126.70	2.9	128.07	3.6
0.15	128.51	2.2	127.17	4.1	128.44	2.1	130.47	3
0.2	130.17	1	128.65	3.8	125.83	1.2	131.74	3.3
0.25	131.66	1.6	129.75	2.8	126.84	2	131.90	3.1
0.3	131.26	1.9	127.96	2.3	126.73	2.3	132.33	3.3
0.35	132.82	1.9	129.07	2.8	125.86	2.1	132.75	2.9
0.4	134.74	1.5	130.27	2.7	124.57	1.7	132.00	2.5

0.45	134.36	2.3	132.64	3.5	125.57	1.6	132.01	3.2
0.5	135.65	1.4	131.62	2.2	124.40	1.3	131.77	2.4
0.55	132.68	1.2	133.36	1.5	123.77	1.7	129.93	1.6
0.6	133.91	1.6	131.02	2.4	122.17	1.7	120.51	1.6
0.65	133.81	1.3	131.10	2.2	121.98	2.8	119.55	2.5
0.7	133.45	2.1	133.68	2.3	117.54	2.1	105.77	2.9
0.75	131.89	1.1	135.07	2.7	116.55	3.1	102.03	3
0.8	132.56	2.6	134.02	2.9	112.09	3.3	100.60	3.7
0.85	128.85	1.6	134.05	1.9	108.84	4.2	99.69	4.6
0.9	128.97	3.2	131.35	3.7	105.69	5	97.45	5.4

Table S2. Values of $E_{\alpha j}$ and $\ln A_{\alpha j}$ used in the compensation effect calculations for ILs $[C_4MIM][C_{11}COO]$ and $[C_{10}MIM][C_{11}COO]$.

	$[C_4MIM][C_{11}COO]$		$[C_{10}MIM][C_{11}COO]$	
Kinetic model	$E_{\alpha j}$	$\ln A_{\alpha j}$	$E_{\alpha j}$	$\ln A_{\alpha j}$
P4	-83.9	-25	-58	-17
P3	-75	-22	-47	-14
P2	-56	-18	-26	-9.3
P2/3	54	8.6	98	20
D1	109	22	160	34
F1	121	27	134	31
A4	11	0.03	17	2
A3	23	3	30	5
A2	48	9	56	12
D3	211	46	245	54
R3	81	16	102	21
R2	61	11	86	18
D2	147	31	194	43
F0	-0.21	-4	36.24	5.6

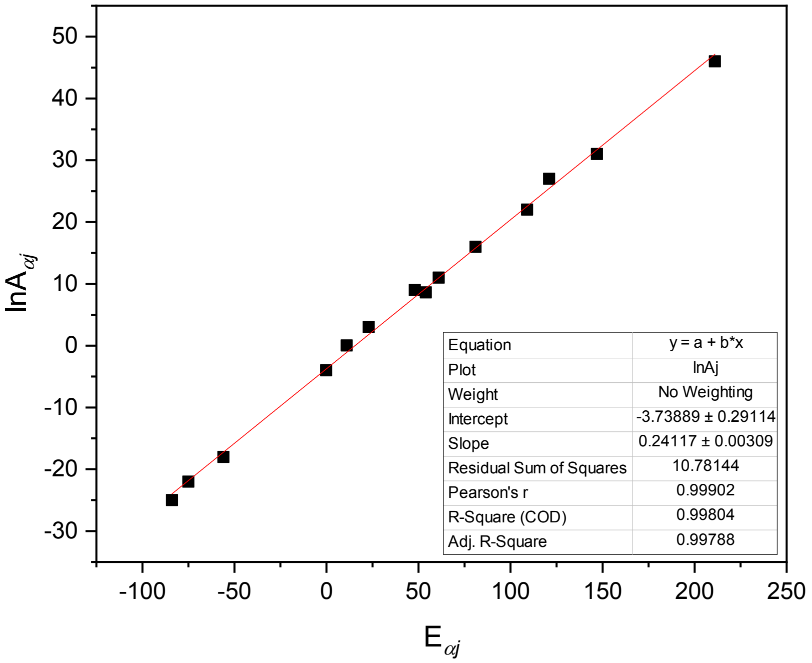

Figure S13. Compensation effect plot for [C_4MIM][C_{11}COO].

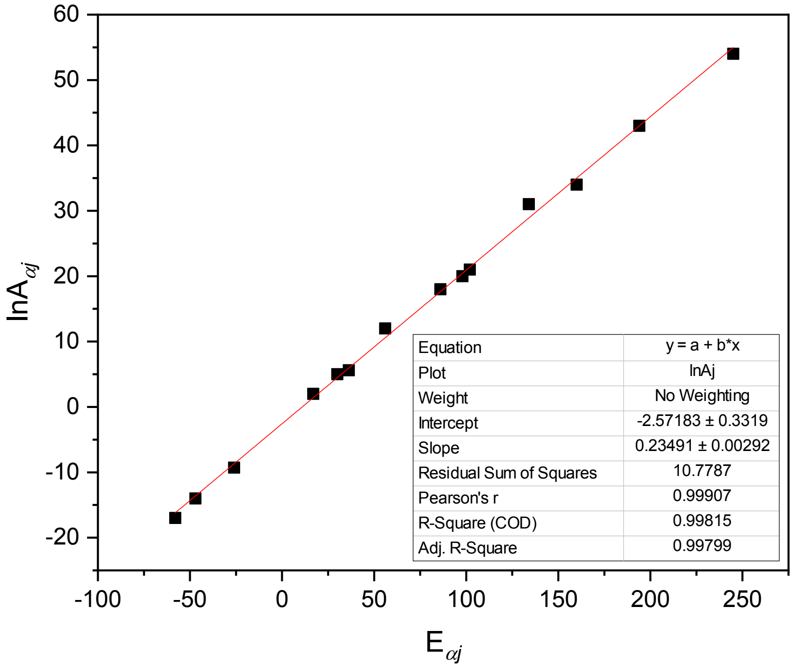

Figure S14. Compensation effect plot for [C_{10}MIM][C_{11}COO].

Table S3. Values of $\ln A_\alpha$ (min^{-1}) for the ILs $[C_4MIM][C_{11}COO]$ and $[C_{10}MIM][C_{11}COO]$ determined using the compensation effect.

	$[C_4MIM][C_{11}COO]$		$[C_{10}MIM][C_{11}COO]$	
α	$\ln A_\alpha$ (min^{-1})	Error (min^{-1})	$\ln A_\alpha$ (min^{-1})	Error (min^{-1})
0.1	26.66	0.61	26.77	0.79
0.15	27.25	0.47	27.30	0.88
0.2	27.65	0.21	27.65	0.81
0.25	28.01	0.33	27.90	0.60
0.3	27.92	0.40	27.48	0.48
0.35	28.29	0.39	27.74	0.59
0.4	28.76	0.32	28.02	0.57
0.45	28.66	0.47	28.58	0.75
0.5	28.98	0.30	28.34	0.46
0.55	28.26	0.26	28.75	0.33
0.6	28.56	0.33	28.20	0.51
0.65	28.53	0.26	28.22	0.46
0.7	28.45	0.45	28.83	0.49
0.75	28.07	0.24	29.15	0.57
0.8	28.23	0.54	28.91	0.62
0.85	27.34	0.33	28.91	0.40
0.9	27.36	0.67	28.28	0.80
